# Engineering Catalytic CoSe–ZnSe Heterojunctions Anchored on Graphene Aerogels for Bidirectional Sulfur Conversion Reactions

**DOI:** 10.1002/advs.202103456

**Published:** 2021-10-27

**Authors:** Zhengqing Ye, Ying Jiang, Tianyu Yang, Li Li, Feng Wu, Renjie Chen

**Affiliations:** ^1^ Beijing Key Laboratory of Environmental Science and Engineering School of Material Science & Engineering Beijing Institute of Technology Beijing 100081 China; ^2^ Advanced Technology Research Institute Beijing Institute of Technology Jinan 250300 China; ^3^ Collaborative Innovation Center of Electric Vehicles in Beijing Beijing 100081 China

**Keywords:** bidirectional electrocatalysts, CoSe–ZnSe heterojunctions, graphene aerogels, lithium–sulfur batteries, sulfur conversion

## Abstract

Sluggish sulfur reduction and lithium sulfide (Li_2_S) oxidation prevent the widespread use of lithium–sulfur (Li–S) batteries, which are attractive alternatives to Li−ion batteries. The authors propose that a transition metal selenide heterojunction (CoSe–ZnSe) catalytically accelerates bidirectional sulfur conversion reactions. A combination of synchrotron X‐ray absorption spectroscopy and density functional theory calculations show that a highly active heterointerface with charge redistribution and structure distortion effectively immobilizes sulfur species, facilitates Li ion diffusion, and decreases the sulfur reduction and Li_2_S oxidation energy barriers. The CoSe–ZnSe catalytic cathode exhibits high areal capacities, good rate capability, and superior cycling stability with capacity fading rate of 0.027% per cycle over 1700 cycles. Furthermore, CoSe–ZnSe heterojunctions anchored on graphene aerogels (CoSe–ZnSe@G) enhance ionic transport and catalytic activity under high sulfur loading and lean electrolyte conditions. A high areal capacity of 8.0 mAh cm^−2^ is achieved at an electrolyte/sulfur ratio of 3 µL mg^−1^. This study demonstrates the importance of bidirectional catalytic heterojunctions and structure engineering in boosting Li–S battery performances.

## Graphical abstract

A highly active transition metal selenide heterojunction (CoSe–ZnSe) is presented as a bidirectional catalyst for lithium–sulfur batteries. The improved trapping–diffusion–conversion for polysulfides redox afforded by the charge redistributed heterointerface is demonstrated by synchrotron X‐ray absorption spectroscopies and first‐principles calculations. Furthermore, CoSe–ZnSe heterojunctions anchored on graphene aerogels are designed to maximize the catalytic conversion of polysulfides under lean‐electrolyte operation.

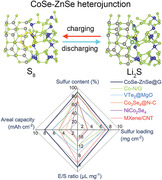

## 1 Introduction

Sulfur (S_8_) is an attractive electrode material with low cost and excellent ability to store lithium (1675 mAh g^−1^); hence the great potential for use in electric vehicles and grid‐level energy storage.^[^ ^1^, ^2^, ^3^ ^]^ However, an inherent problem of sulfur electrode is the sluggish reaction kinetics associated with the complex polysulfides (LiPS) transformations and multistep electron‐transfer processes.^[^ ^4^, ^5^ ^]^ The slowness of the sulfur conversion reaction leads to accumulation of soluble LiPS on the cathode side and their loss to the electrolyte by migration. The irreversible reactions between the LiPS intermediates and the lithium metal also cause degradation of the lithium metal anode. The depressed deposition and oxidation of solid lithium sulfide (Li_2_S) on cathode results in loss of active sulfur species and ion/electron transportation.^[^ ^6^ ^]^ Kinetically unfavorable cathode reactions therefore seriously limit rate capabilities and cycling performances of lithium–sulfur (Li–S) batteries.

The development of techniques for accelerating sulfur redox reactions via electrocatalysis is urgently requested.^[^ ^7^, ^8^ ^]^ Many of the transition‐metal‐based electrocatalysts reported to date are metals such as Co,^[^ ^9^ ^]^ metal oxides,^[^ ^10^ ^]^ metal sulfides,^[^ ^11^, ^12^ ^]^ and metal phosphides,^[^ ^13^, ^14^ ^]^ which also adsorb LiPS intermediates. The deployment of these catalysts enables restriction of LiPS shuttling and promotion of sulfur transformation reactions. Although good progress has been made, battery performances are still unsatisfactory because a single catalyst does not provide the comprehensive improvement of the redox reactivity in terms of factors such as LiPS adsorption, ion diffusion, and electron transfer in a Li–S chemistry. The formation of heterojunctions by constructing dissimilar coupling nanocrystals with different bandgaps for LiPS transformation could meet the above requirements. Recently, it has been reported that heterostructured catalytic cathodes, such as VTe_2_@MgO,^[^ ^15^ ^]^ MoN–VN,^[^ ^16^ ^]^ NiO–NiCo_2_O_4_,^[^ ^17^ ^]^ and TiO_2_–Ni_3_S_2_^[^ ^18^ ^]^ have synergistic functions, namely strong LiPS immobilization on the adsorption sites and rapid electron transfer on the conductive sites. Heterostructure engineering therefore provides opportunities for constructing Li–S batteries with high rate and long cycle life. Enhanced Li–S battery performances are generally attributed to the chemical immobilization abilities of polar metal compounds and fast LiPS conversion of conductive component counterparts. The fundamental mechanism of the acceleration of LiPS transformation by phase interfaces with heterogeneous electronic states is still unclear. Moreover, previous reports have suggested that heterostructured sulfur cathodes possessed limited surface area and insufficient catalysis sites for two‐directional sulfur conversion, which leads to low sulfur utilization and sulfur loading.

In recent years, transition‐metal selenides (TMSes) have drawn increasing attention for use in energy storage and conversion fields.^[^ ^19^, ^20^, ^21^ ^]^ Zhang et al. prepared urchin‐shaped NiCo_2_Se_4_ nanostructure for use as sulfur hosts for long‐life and high‐rate Li–S batteries.^[^ ^22^ ^]^ A highly polar and conductive bimetallic selenide gave enhanced capture of soluble LiPSs, fast electron transfer, and a catalytic enhancement of the redox kinetics. Chen and coworkers recently reported that a highly efficient CoSe electrocatalyst with a hierarchical porous nano‐polyhedron (CS@HPP) structure can promote LiPSs capture/diffusion and deposition/oxidation of Li_2_S.^[^ ^23^ ^]^ Excellent electrochemical performances were synchronously achieved for both slurry‐bladed and freestanding sulfur cathodes. In addition, TMSe heterojunctions, for example, MoSe_2_–NiSe and CoSe_2_–MoSe_2_, are efficient electrocatalysts in hydrogen evolution.^[^ ^24^, ^25^ ^]^ Phase interfaces induced by TMSe heterojunctions can lower the activation barriers and give higher catalytic activities, and therefore improve reaction kinetics and electrochemical performance. The design of TMSe heterojunctions is therefore important for further enhancing Li–S battery performances, but reports of such heterointerfaces are rare. Moreover, these TMSes are generally limited to the single nanoparticle forms, and are not sufficiently efficient and robust for the sulfur conversion reactions under a lean electrolyte condition.

In this work, a heterointerface for boosting sulfur redox reaction was obtained by constructing a TMSe heterojunction. Density functional theory (DFT) calculations were performed to prove that TMSe heterointerface with metallic properties decreases the Gibbs energy barriers to sulfur reduction and Li_2_S decomposition. Experimental results confirmed that the TMSe heterointerface catalytically accelerates sulfur reduction and Li_2_S oxidation during discharge/charge processes. Enhanced bidirectional sulfur conversion enabled a CoSe–ZnSe/S catalytic cathode to achieve an outstanding capacity of 1654 mAh g^−1^ at 0.1 C and maintain 808 mAh g^−1^ at 3 C. Long‐term cyclic stability with a low capacity decay of 0.027% per cycle after 1700 cycles at 2 C was achieved. Furthermore, CoSe–ZnSe heterojunctions anchored on 3D graphene aerogels (CoSe–ZnSe@G) derived from the metal–organic framework (MOF)@graphene aerogel composites were synthesized. The 3D macroporous interconnected carbon network maximized catalytic site exposure and greatly enhanced mass transport and sulfur species conversion during cycling under lean electrolyte conditions. A high areal capacity, that is, 8.0 mAh g^−1^, was achieved under high sulfur loading of 7.7 mg cm^−2^ and low electrolyte/sulfur (E/S) ratio of 3 µL mg^−1^.

## 2 Results and Discussion

**Figure 1:**
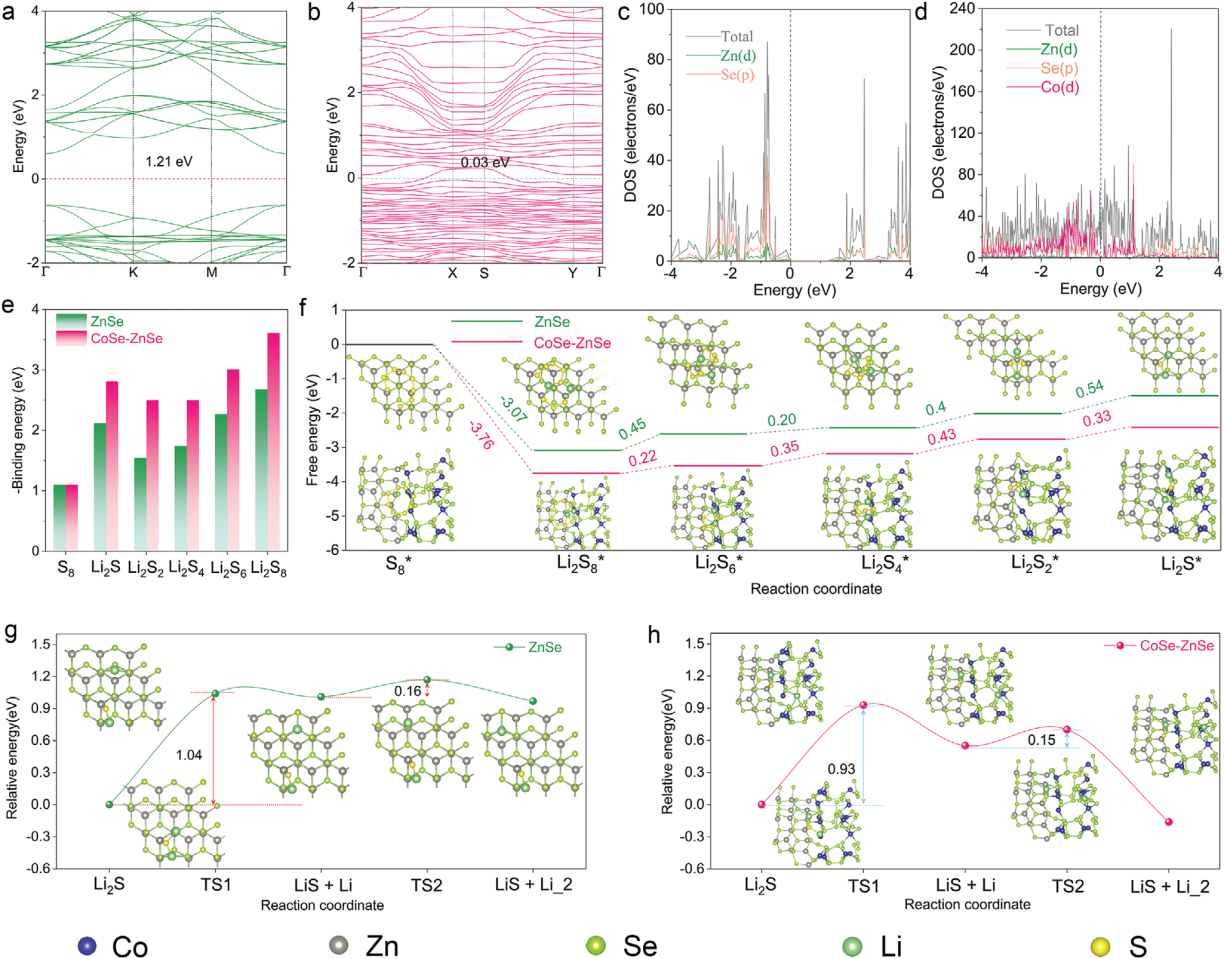
The band structure of a) ZnSe and b) CoSe–ZnSe. The density of states of c) ZnSe and d) CoSe–ZnSe. e) Calculated adsorption energy of sulfur species with ZnSe and CoSe–ZnSe heterointerface. f) Relative free energy for the reduction S_8_ to Li_2_S on the ZnSe and CoSe–ZnSe heterointerface (insets: the optimized adsorption structures of sulfur species on ZnSe and CoSe–ZnSe substrate). Energy profiles of the decomposition barriers of Li_2_S and lithium ion diffusion on the g) ZnSe and h) CoSe–ZnSe heterointerface (insets: the corresponding decomposition of Li_2_S and lithium ion diffusion pathways for ZnSe and CoSe–ZnSe).

DFT calculations were performed to investigate the enhancement of bidirectional sulfur conversion on the CoSe–ZnSe heterointerface at the atomic level. As shown in Figure S1, Supporting Information, two models, namely single metal selenide (ZnSe) and CoSe–ZnSe heterointerface were considered in our simulation. The energy band structure of ZnSe have a wide bandgap of 1.21 eV, indicating low electronic conductivity (**Figure** **1**a). In contrast, the ZnSe–CoSe heterointerface possesses a negligible energy bandgap (Figure 1b), which reflects its metallic character. The calculated density of states of CoSe–ZnSe shows no gap of states at the Fermi level (Figure 1d), and a bandgap of 1.21 eV for ZnSe (Figure 1c). This further indicates that the ZnSe–CoSe heterojunction possesses intrinsic conductivity. Figures S2 and S3, Supporting Information, show the optimized adsorption configurations of LiPSs with ZnSe and CoSe–ZnSe surfaces; Li^+^ and S_n_^2−^ bind with the Se and Zn atoms on the ZnSe surface. At the CoSe–ZnSe heterointerface, Li^+^ binds with Se atoms and S_n_^2−^ binds with Co and Zn atoms. The calculated results show that the binding energies of LiPS at six different lithiation stages on the CoSe–ZnSe heterointerface are all higher than those on the ZnSe surface (Figure 1e). The enhanced electrical conductivity and binding strength endowed by the TMSes heterointerface promote bidirectional conversion reactions of S_8_ and Li_2_S.

The sulfur reduction pathways on the ZnSe and CoSe–ZnSe surface were investigated. The optimized structures of the reactive intermediates and their relative free energy profiles are shown in Figure 1f. The data clearly show that the conversion from S_8_ to Li_2_S_8_ is exothermic and the following four steps, which involve the transformations of Li_2_S_6_, Li_2_S_4_, Li_2_S_2_, and Li_2_S, are endothermic. Specifically, the Gibbs free energy from S_8_ to Li_2_S_8_ on the CoSe–ZnSe heterojunction surface is more spontaneously exothermic than that on the ZnSe surface. The energy barriers for the last two reduction steps (the formation of Li_2_S_2_ from Li_2_S_4_ and the formation of Li_2_S from Li_2_S_2_) are larger than those of the other steps. This indicates that the Li_2_S_2_/Li_2_S deposition is the rate‐determining step during the discharge process. The largest Gibbs free energies for the CoSe–ZnSe and ZnSe are 0.43 eV (from Li_2_S_4_ to Li_2_S_2_) and 0.54 eV (from Li_2_S_2_ to Li_2_S), respectively. The lower energy barriers on the CoSe–ZnSe heterojunction suggest that the sulfur reduction is thermodynamically more favorable on this interface than on ZnSe.

Li_2_S decomposition is the first step in the charging process. The overall Li_2_S decomposition consists of two steps: a single Li ion dissociates from Li_2_S and the dissociated Li^+^ diffuses away from the LiS cluster.^[^ ^26^ ^]^ Figure 1g,h shows the energy profiles for the Li_2_S decomposition processes on the ZnSe and CoSe–ZnSe surfaces. The Li_2_S dissociation energies on the both substrates are much larger than the Li ion diffusion barriers, suggesting that the breaking of the Li—S bond is the rate‐limiting step. The calculated dissociation energy barrier of Li_2_S on CoSe–ZnSe heterointerface (0.93 eV) is smaller than that on ZnSe (1.04 eV). This indicates that heterointerface in the CoSe–ZnSe acts as a catalytic center for acceleration of the phase transformation of Li_2_S during charging process. The CoSe–ZnSe heterointerface maintains a smaller lithium diffusion barrier compared with that of ZnSe, which facilitates the subsequent conversion reactions after Li_2_S decomposition.

**Figure 2:**
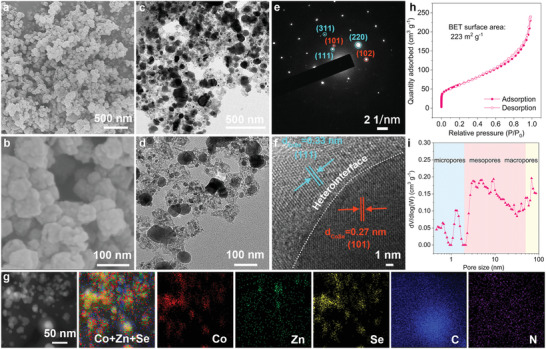
a,b) FESEM images, c,d) TEM image, e) SAED diffraction pattern, f) HRTEM image, g) HAADF‐STEM image and corresponding EDS elemental mapping of Co, Zn, Se, C, and N, h) N_2_ physisorption isotherms, and i) pore size distribution of CoSe–ZnSe.

The theoretical results show that a rationally designed CoSe–ZnSe heterojunction serves as a catalytic cathode and enables the construction of high‐capacity, high‐rate, and long‐life Li–S batteries. A Co and Zn bimetallic MOF (Co/Zn‐MOFs) precursor with a smooth surface and high crystallinity was synthesized by a facile and scalable coprecipitation method at room temperature (Figure S4, Supporting Information). Field emission scanning electron microscopy (FESEM) images exhibited good retention of porous polyhedron structure during conversion of the Co/Zn‐MOF precursor by in situ carbonization–selenization (**Figure** **2**a,b). Transmission electron microscopy images showed that calcination converted Co/Zn‐MOFs into the CoSe–ZnSe nanoparticles embedded in porous N‐doped carbon networks (Figure 2c,d). For comparison, ZnSe was synthesized from Zn‐MOF (Figures S5 and S6, Supporting Information). X‐ray diffraction (XRD) pattern exhibited distinct peaks from *P*63/*mmc* CoSe (PDF no. 70‐2870) and *F*‐43*m* ZnSe (PDF no. 88‐2345) phase and confirmed the presence of both phases in the CoSe–ZnSe structures (Figure S7a, Supporting Information). Compared with those of CoSe (blue line) and ZnSe (green line), the positive shift and the decreased crystallinity of the peaks for CoSe–ZnSe (red line) (Figure S7b,c, Supporting Information) may be ascribed to the increased deformation and disorder in the crystal structure.^[^ ^27^, ^28^ ^]^

X‐ray photoelectron spectroscopy (XPS) measurements further affirmed the presence of Co, Zn, Se, C, and N (Figure S8, Supporting Information). A selected‐area electron diffraction pattern showed the presence of the (101) and (102) planes of CoSe and (111), (220), and (311) crystal planes of ZnSe (Figure 2e). This confirms the formation of a CoSe–ZnSe heterojunction. High‐resolution transmission electron microscopy (HRTEM) was used to examine the twinborn interfaces between the two components (Figure 2f and Figure S9, Supporting Information). The calculated the interface density value of the CoSe–ZnSe is determined to be 0.73 × 10^8^ m^−1^ based on the experimental HRTEM image (Figure 2f).^[^ ^29^ ^]^ The lattice fringes of 0.27 and 0.33 nm were consistent with the (101) plane of CoSe and (111) plane of ZnSe, respectively. The presence of a heterojunction was confirmed by high‐angle annular dark‐field scanning transmission electron microscopy, and corresponding energy‐dispersive spectroscopy (EDS) elemental mapping analysis showed the homogeneous distribution of Co, Zn, and Se across the examined zone (Figure 2g).

The Raman spectra of CoSe–ZnSe (Figure S10, Supporting Information) exhibits peaks at 138, 198, and 246 cm^−1^, which correspond to ZnSe,^[^ ^30^ ^]^ and peaks at about 467, 513, and 669 cm^−1^, which correspond to CoSe. The results demonstrate the coexistence of CoSe and ZnSe in this CoSe–ZnSe hybrid. The red‐shift in characteristic peaks of CoSe–ZnSe heterojunction further confirms the strong interaction between ZnSe and CoSe at the heterointerfaces, which brings about a strong out‐of‐plane vibration in the force of internal built‐in *E*‐field.^[^ ^31^ ^]^ Moreover, the similar ratio of the intensity of peak D to that of peak G for CoSe–ZnSe, CoSe, and ZnSe indicates similar disordered graphite‐like carbon structures in these three hybrids.^[^ ^32^ ^]^ This enables the exclusion of the influence of carbon skeleton on electrochemical behavior, allowing us to concentrate instead on the effect of CoSe–ZnSe heterojunction.

The N_2_ adsorption–desorption isotherm (Figure 2h and Figure S11, Supporting Information) exhibits a steep N_2_ uptake at low relatively pressure (*P*/*P*_0_ = 0–0.005) and a hysteresis loop at high relatively pressure (*P*/*P*_0_ = 0.45–0.98), indicating the coexistence of micropores and mesopores.^[^ ^33^ ^]^ The specific surface area (223 m^2^ g^−1^) and the pore volume (0.37 cm^3^ g^−1^) of CoSe–ZnSe are higher than those of ZnSe (Table S1, Supporting Information). The surface area value of CoSe–ZnSe is much larger than those of recently reported heterostructures (Table S3, Supporting Information), and this facilitates uniform deposition of active sulfur species and provides abundant surface‐active sites for LiPS adsorption and transformation. The pore size distribution (Figure 2i) of CoSe–ZnSe exhibits abundant micropores of size 0.6–1.3 nm, small mesopores of size 3.1–9.3 nm, large mesopores of size 27–41 nm, and macropores of size 50–68 nm. The micropores structure provides the sustainable adsorption of electrolyte and soluble LiPS molecules, which enables the sufficient wetting of reaction and catalytic sites of heterointerface, and therefore the fast solid–liquid conversion kinetics. The small mesopores ensure the confined LiPS adsorption sites and effectively accelerate ion/electron transport during the electrocatalytic process.^[^ ^34^ ^]^ Actually, more sulfur can be filled in the larger mesoporous voids and macropores.^[^ ^6^, ^34^ ^]^ Thermogravimetric analysis (TGA) analysis revealed that the contents of CoSe–ZnSe heterojunction content in the hybrid was about 84.3 wt% (Figure S12, Supporting Information).

**Figure 3:**
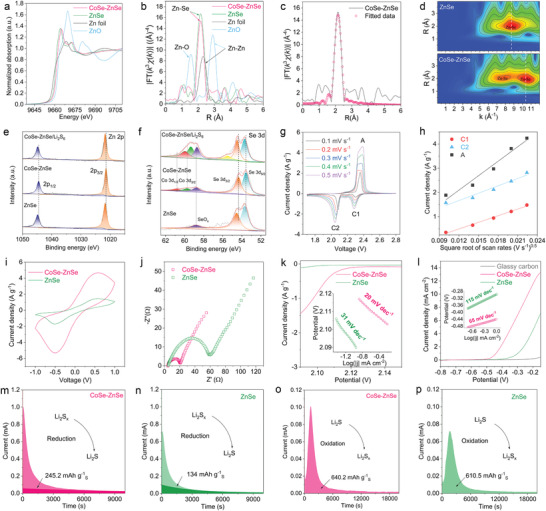
a,b) XANES spectra and FT‐EXAFS spectra at the Zn K‐edge for the CoSe–ZnSe, ZnSe, ZnO, and Zn powders. c) FT‐EXAFS fitting curves of the CoSe–ZnSe at Zn K‐edge. d) WT‐EXAFS spectra of the CoSe–ZnSe and ZnSe. e) Zn 2p and f) Se 3d XPS spectrums of ZnSe, CoSe–ZnSe, and CoSe–ZnSe/Li_2_S_6_. g) CV curves of the CoSe–ZnSe/S electrode at various voltage scan rates. h) Plot of CV peak current versus square root scan rates for CoSe–ZnSe/S electrode. i) CV and j) EIS of Li_2_S_6_ symmetric cells. k) CV curves and C2 reduction peak derived Tafel plots as inset. l) LSV curves with the derived Tafel plots as inset. m,n) Precipitation profiles of Li_2_S. o,p) The dissolution profiles of Li_2_S.

Synchrotron X‐ray absorption near‐edge structure (XANES) and extended X‐ray absorption fine structure (EXAFS) spectroscopies were used to investigate the local atomic and electronic structure of the ZnSe–CoSe. As shown in **Figure** **3**a, Zn K‐edge XANES curves of ZnSe–CoSe and ZnSe show their edge energy between those of the metallic Zn foil (Zn^0^) and the ZnO (Zn^2+^), suggesting the positive charge Zn species with the average valence state between 0 and +2. The half‐edge energy of the ZnSe–CoSe shifted to a slightly higher energy than that of the ZnSe. In addition, the depressed white‐line peak intensity is obviously observed for ZnSe–CoSe. These results suggest the intense electronic interactions between CoSe and ZnSe domains.^[^ ^29^, ^35^ ^]^ The Fourier‐transform (FT) EXAFS spectra of the Zn K‐edge show a main peak around 2.45 Å, corresponding to the Zn—Se bond in CoSe–ZnSe (Figure 3b,c). Moreover, the Zn coordination number in CoSe–ZnSe is lower than that in ZnSe (Table S2, Supporting Information), which could be attributed to the coexistence of heterogeneous spin states in the heterointerface and the mismatch in the degree of strong Jahn–Teller distortion.^[^ ^20^ ^]^ In wavelet transform (WT) EXAFS contour plots, ZnSe has the maximum intensity at *k* = 8.7 Å^−1^, while the maximum intensity for CoSe–ZnSe presents is at *k* = 10.4 Å^−1^ (Figure 3d). The obvious shift in the WT maximum further demonstrates the different coordination environments and structural disorders in CoSe–ZnSe.

The valence state of the CoSe–ZnSe was also studied by XPS measurements. Driven by the built‐in internal *E*‐field resulted from the heterojunction, the CoSe–ZnSe Zn 2p and Se 3d peaks exhibited slight negative shifts relative to those of ZnSe, emphasizing the electron cloud bias from CoSe side to ZnSe side (Figure 3e,f).^[^ ^36^ ^]^ Moreover, the existence of Co 3p and SeO*_x_* is attributed to the Co—Se bond and the oxide species of Se on the CoSe–ZnSe surfaces (Figure 3f).^[^ ^37^ ^]^ Further understanding the chemical interaction between LiPS and heterointerface is the first step in achieving accelerated sulfur species transformations. The digital images in Figure S13, Supporting Information, shows that the color of a solution containing CoSe–ZnSe is lighter than that of a ZnSe solution, which demonstrates better adsorption at the heterojunction. XPS was then used to investigate the chemical interaction between LiPS and CoSe–ZnSe. As shown in Figure 3e, the Zn 2p_1/2_ and Zn 2p_3/2_ peaks at around 1021.4 and 1044.5 eV in the fitted XPS curves of pristine CoSe–ZnSe shift to 1021.7 and 1044.8 eV, respectively, after the adsorption of Li_2_S_6_. Similarly, all the Co 2p_3/2_ and 2p_1/2_ peaks detected in LiPS‐treated CoSe–ZnSe shifted to higher binding energies (Figure S14, Supporting Information). The changes in the peak position in the Zn 2p and Co 2p spectra can be attributed to interactions between Co/Zn heterointerface sites and surrounding electronegative sulfur species. Moreover, the Se 3d_3/2_ and Se 3d_5/2_ peaks in the CoSe–ZnSe/LiPSs shifted to higher values, and a new peak arose at 55.5 eV, which indicates the existence of Li—Se bonds (Figure 3f).^[^ ^26^ ^]^ The formation of a sulfiphilic and lithiophilic heterointerface can therefore greatly enhance LiPS retention in the cathode and suppress LiPS diffusion to Li metal anode.

Sulfur was incorporated into CoSe–ZnSe (CoSe–ZnSe/S) by a melt‐diffusion method (see the Experimental Section in Supporting Information for details). XRD analysis confirmed the presence of crystalline sulfur in the CoSe–ZnSe heterostructure and the retention of the CoSe–ZnSe crystal structure (Figure S15a, Supporting Information). TGA showed that the sulfur content of the CoSe–ZnSe/S electrode was 66.2 wt% (Figure S15b, Supporting Information). The structure of the obtained product resembles the original structure, and sulfur is uniformly distributed throughout CoSe–ZnSe (Figures S16–S18, Supporting Information). ZnSe with the similar sulfur content (ZnSe/S) was also prepared by the same procedure (Figure S19a,b, Supporting Information). The ZnSe/S consists of agglomerated and unevenly distributed particles (Figures S19c,d and S20, Supporting Information). N_2_ physisorption analysis also indicated the successful filling of the porous structure by sulfur (Figure S21, Supporting Information). Cyclic voltammetry (CV) were performed at different scan rates (Figure 3g and Figure S22a, Supporting Information) to investigate the reaction kinetics and Li ion diffusion in the electrodes. Two cathodic peaks (C1 and C2) were identified during reduction of S_8_ into soluble Li_2_S*_x_* (4 ≤ *x* ≤ 8) and subsequent transformation to insoluble Li_2_S_2_/Li_2_S, respectively. The anodic peak (A) accounts for the one‐step oxidation conversion of Li_2_S_2_/Li_2_S to sulfur. Figure 3h shows that all peak currents have a linear relationship with the square root of scan rates, and the Li diffusion properties can be evaluated by using the classical Randles–Sevcik equation:

(1) $$I_{p}=\left(2.69\times 10^{5}\right)\cdot n^{1.5}\cdot A\cdot D^{0.5}\cdot C_{\mathrm{Li}}\cdot \upsilon^{0.5}$$

where *I*_p_ is the peak current density, *n* is the reaction electrons number, *A* is the electrode area, *D* is the Li ion diffusion coefficient, *C*_Li_ is the Li‐ion concentration, and *υ* is the sweep rate. The curve slopes have a positive correlation with the Li ion diffusion performance. This indicates that CoSe–ZnSe/S gives faster Li ion diffusion and better reaction kinetics than the ZnSe/S electrode during bidirectional sulfur conversion processes (Figure S22b–d, Supporting Information).

Symmetric Li_2_S_6_ cells were designed and used to better understand transformation kinetics of LiPS. As shown in Figure 3i, the current density of CoSe–ZnSe is obviously higher than that of ZnSe, which indicates heterointerface catalytically accelerates sulfur conversion reactions. A low charge transfer resistance (*R*_ct_) was verified by electrochemical impedance spectroscopy (EIS), implying significant improvement of sulfur redox kinetics by CoSe–ZnSe heterointerface (Figure 3j). The Tafel slopes derived from the cathodic CV peak (C2) for different cathodes are shown in Figure 3k. The Tafel slope for the CoSe–ZnSe heterojunction is lower than that for ZnSe, indicating the boosted Li_2_S_2_/Li_2_S deposition during sulfur reduction process. Three‐electrode linear sweep voltammetry tests were performed to study the Li_2_S oxidization (Figure 3l). The CoSe–ZnSe heterojunction current density is much higher and the onset potential is much lower (−0.51 vs −0.42 V) than those of ZnSe, indicating the smaller energy barrier for Li_2_S oxidation process. Enhancement of the Li_2_S oxidation kinetics is supported by the corresponding Tafel plots, which exhibit a smaller Tafel slope (65 mV dec^−1^) for CoSe–ZnSe electrode than for ZnSe (115 mV dec^−1^).

The improved bidirectional sulfur conversion on the CoSe–ZnSe heterointerface was further investigated by precipitation and dissolution measurements of Li_2_S. As shown in Figure 3m,n, the current response and precipitation capacity of CoSe–ZnSe are higher than those of ZnSe, indicating that the CoSe–ZnSe heterojunction greatly promotes Li_2_S nucleation and growth. The morphologies of Li_2_S deposited on different catalyst supports were further observed by SEM. Uniform Li_2_S deposition was observed on carbon paper–CoSe–ZnSe, whereas Li_2_S aggregation clearly occurred on the carbon paper–ZnSe surface (Figure S23a,b, Supporting Information). For the Li_2_S dissolution process, CoSe–ZnSe gives a much higher dissolution current response, earlier dissolution time, and larger dissolution capacity than does ZnSe (Figure 3o,p). In addition, bulky agglomerated Li_2_S deposits were still present on the carbon paper–ZnSe surface after reaction, while Li_2_S deposits on the carbon paper–CoSe–ZnSe surface almost disappeared (Figure S23c,d, Supporting Information). These results are in accord with the calculation results, which showed that CoSe–ZnSe heterointerface boosts bidirectional sulfur conversion reactions.

**Figure 4:**
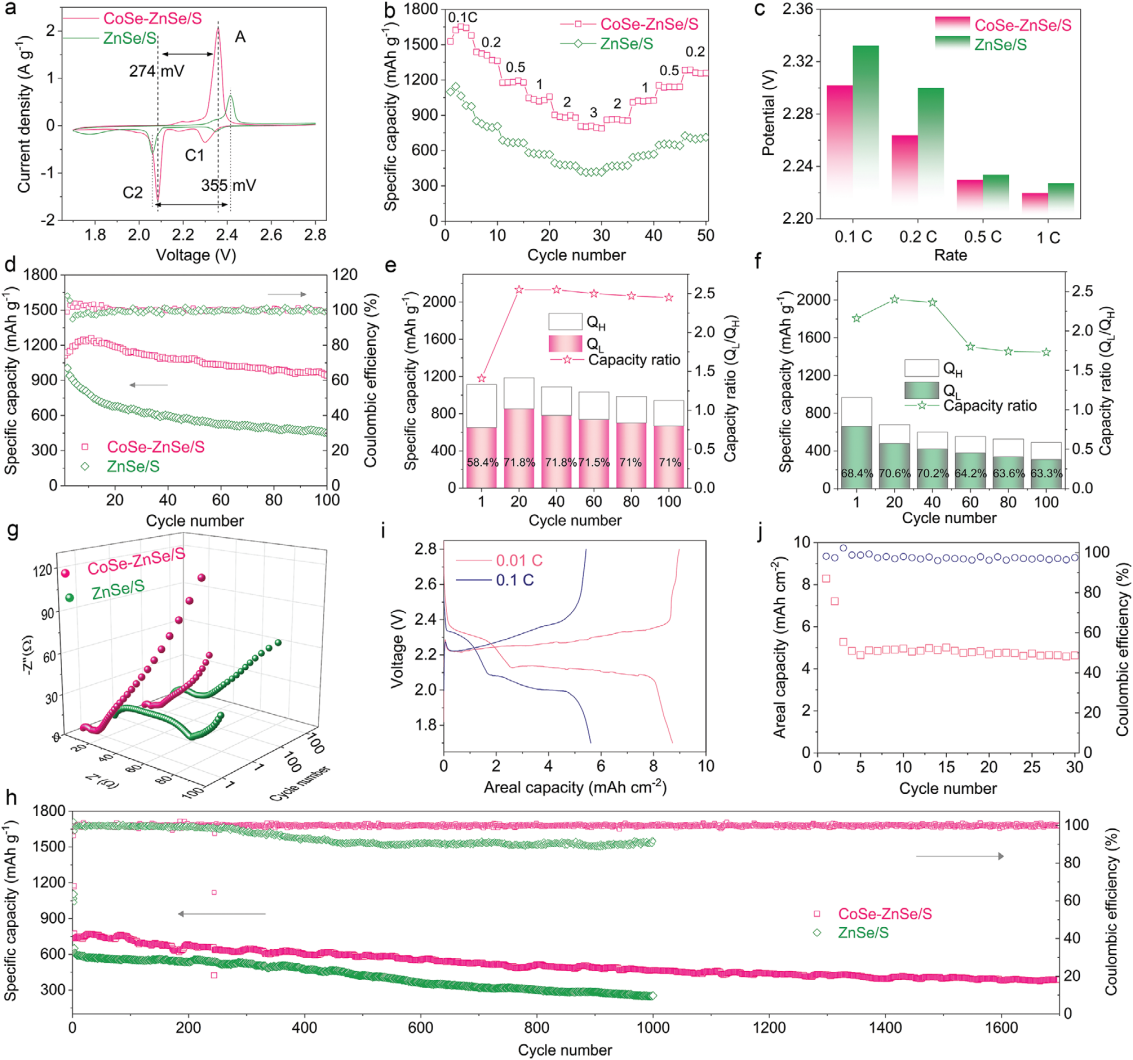
a) CV curves at 0.1 mV s^−1^, b) rate capabilities, c) the initial charge potential of the different C rates, and d) cycling performance at 0.2 C for CoSe–ZnSe/S and ZnSe/S cathodes. Capacity contributions of *Q*_H_ and *Q*_L_ and the *Q*_L_/*Q*_H_ ratios of the representative cycles for the e) CoSe–ZnSe/S and f) ZnSe/S cathodes. g) EIS of different cathodes before cycling and after 100 cycles at 0.2 C. h) Long‐term cycling performances at 2 C for CoSe–ZnSe/S and ZnSe/S cathodes. i) Charge–discharge profiles at various C rates. j) Cycling performances at 0.1 C of CoSe–ZnSe/S electrodes with a high sulfur loading.

Electrochemical evaluations were performed to identify the advantageous catalysis effects of CoSe–ZnSe heterointerface on Li–S batteries. We first verified that the discharge capacities of CoSe–ZnSe, CoSe, and ZnSe catalysts are very low (Figure S24a–c, Supporting Information), and thus have an almost negligible contribution to the total discharge capacity of sulfur electrodes. Furthermore, based on the CV measurements, CoSe–ZnSe, CoSe, and ZnSe catalysts remain electrochemically inert within voltage range of 1.7–2.8 V versus Li/Li^+^ (Figure S24d–f, Supporting Information). **Figure** **4**a shows that in the CV curves the separation between the C2 and A peaks are 274 and 355 mV for the CoSe–ZnSe/S and ZnSe/S, respectively. This demonstrates polarization reduction and redox kinetics enhancement. The positive shift in the C2 peak, negative shift in the A peak, and significantly increased current density in the CoSe–ZnSe electrode indicate the higher catalytic activity of the heterointerface sites in the transformation reaction of sulfur species. The efficient catalysis transformation of CoSe–ZnSe enables the battery to deliver the highest capacity of 1654 mAh g^−1^ (the sulfur utilization rate of 98.7%) at 0.1 C, and a reversible discharge capacity of 808 mAh g^−1^ can be achieved at 3 C (Figure 25a, Supporting Information). When the current rate was changed back to 0.2 C, a discharge capacity of 1258 mAh g^−1^ was recovered (Figure 4b). The rate capability of CoSe–ZnSe/S is much better than those of ZnSe/S (Figure S25b, Supporting Information) and most of the previously reported materials (Table S3, Supporting Information). Figure 4c exhibits that the smallest charge potential at an increasing current rate was obtained for CoSe–ZnSe/S, which indicates faster oxidation of Li_2_S than in the ZnSe/S system.^[^ ^38^ ^]^

The cycling stabilities of the CoSe–ZnSe/S and ZnSe/S are shown in Figure 4d. The reversible capacity at 0.2 C of CoSe–ZnSe/S was 1260 mAh g^−1^, and the corresponding capacity retention was 74.8% after 100 cycles. By contrast, ZnSe/S delivered a lower capacity retention of 45.1%. The superior capacity characteristics of the CoSe–ZnSe host were investigated by extracting the high and low plateau discharge capacities (denoted as *Q*_H_ and *Q*_L_) from the discharge voltage profiles. Figure 4e,f shows the total discharge capacity, the capacity contributions in the high and low plateaus, and the ratio of the capacity in the low plateau to that in the high plateau (*Q*_L_/*Q*_H_) in representative cycles. CoSe–ZnSe/S maintained a high *Q*_L_/*Q*_H_ ratio of 2.45 at 100 cycles. In contrast, ZnSe/S gave a low *Q*_L_/*Q*_H_ ratio of 1.63. CoSe–ZnSe/S maintained a stable contribution to the low plateau capacity, which involved a sluggish reduction of liquid LiPS to solid Li_2_S. This indicates that CoSe‐ZnSe can effectively catalyze LiPS reduction reaction during the discharging process. EIS measurements were performed to further validate the accelerated redox kinetics of sulfur species in the CoSe–ZnSe heterointerface. CoSe–ZnSe/S exhibited lower *R*_ct_ in the high frequency and higher slope in the low frequency region than the ZnSe/S, both before and after cycles. This indicates fast sulfur species transformation and Li^+^ diffusion (Figure 4g).^[^ ^39^ ^]^ Enhanced LiPS transformation was further verified by the postmortem examination of the cycled batteries. A uniform surface shape and structure were observed in CoSe–ZnSe/S cathode (Figure S26a,b, Supporting Information), whereas aggregates of solid sulfur species clearly accumulated on the ZnSe/S electrode surface (Figure S26c,d, Supporting Information). The CoSe–ZnSe heterojunction is also advantageous in Li metal protection. The Li metal anode from the CoSe–ZnSe/S cell retained a smooth surface and intact section morphology (Figure S27a–c, Supporting Information). For the ZnSe/S cell, Li metal anode was almost pulverized or broken into particles with large corrosion thickness because of parasitic reactions between Li metal anode and LiPS (Figure S27d–f, Supporting Information).

Ultralong‐term cycling was performed at high current rate (Figure 4h). ZnSe/S maintained 254 mAh g^−1^ for 1000 cycles at 2 C with a capacity decay of 0.061% per cycle. In contrast, The CoSe–ZnSe/S electrode exhibited excellent reversible capacity of 774 mAh g^−1^ and ultralow capacity fade of 0.027% per cycle during 1700 cycles at 2 C. This cycling stability is better than those achieved in recently reported studies (Table S3, Supporting Information). A CoSe and ZnSe hybrid material (CoSe/ZnSe) without heterojunction was also prepared by simple physically mixing (Figure S28, Supporting Information). The cycling performances of the CoSe/ZnSe without heterojunction sulfur cathode were inferior to those of the CoSe–ZnSe heterojunction sulfur cathodes (Figure S29a,b, Supporting Information). The CoSe–ZnSe heterojunction sulfur electrode exhibits lower overpotentials than that of the CoSe/ZnSe without heterojunction sulfur cathode in both the discharge and charge profiles (Figure S29c, Supporting Information), indicating the lower interfacial energy barriers of the Li_2_S nucleation and decomposition on the surface of CoSe–ZnSe electrode.^[^ ^9^, ^12^ ^]^

Figure S30, Supporting Information, exhibits the rate performance of CoSe–ZnSe/S electrodes with a sulfur loading of 3.0 mg cm^−2^ at various rates between 0.05 and 1.0 C. The corresponding areal capacity values were 3.6, 2.6, 2.1, and 1.8 mAh cm^−2^ at 0.05, 0.2, 0.5, and 1.0 C, respectively. The high areal capacity for CoSe–ZnSe/S is recoverable by switching back to 0.2 C, and subsequent cycling was largely reversible for 108 cycles with a capacity retention of 88.8%. When the CoSe–ZnSe/S electrode thickness was increased to 6 mg cm^−2^, areal capacities correspondingly increase to 5.1 and 4.2 mAh cm^−2^ at 0.05 and 0.2 C, respectively. The longer electron/mass transport distance in this thicker sulfur cathode and the electrolyte‐starved Li–S battery hindered the release of areal capacity when the rate exceeds 0.5 C. When returning to 0.2 C, a highly reversible areal capacity of 4.2 mAh cm^−2^ was recovered. Figure 4i presents a distinct two plateau voltage profile under a higher sulfur loading of 7.1 mg cm^−2^, with a high areal capacity of 8.7 and 5.6 mAh cm^−2^ at 0.01 and 0.1 C, respectively. The CoSe–ZnSe/S cathode achieved the highest areal capacity of a metal‐based heterojunction sulfur host reported to date (Table S3, Supporting Information). A stable cycling performance was displayed by the CoSe–ZnSe/S cathode, with high areal capacity retention value of 4.6 mAh cm^−2^ at a sulfur loading of 4.5 mg cm^−2^ after 30 cycles (Figure 4j).

**Figure 5:**
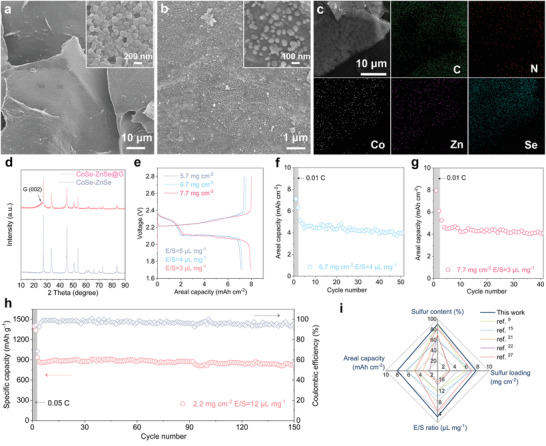
a) SEM images of Co/Zn‐MOFs@G. b) SEM images and c) corresponding EDS images of CoSe–ZnSe@G. d) XRD patterns. e) Charge–discharge profiles at 0.01 C. f,g) Cycling performances at 0.02 C, and h) cycling stability at 1 C of CoSe–ZnSe@G current collector. i) Comparison of the electrochemical performance of recently reported work in Li–S batteries.

In addition to a high sulfur loading (≥6 mg cm^−2^), a low E/S ratio (≤5 µL mg^−1^) is necessary for practical Li–S batteries.^[^ ^40^ ^]^ In these harsh conditions, the aggregation and structural collapse of MOF‐derived nanoparticles would happen during discharging/charging process, resulting in sluggish sulfur conversion and poor Li–S battery performance.^[^ ^41^ ^]^ CoSe–ZnSe heterojunctions anchored on graphene aerogels (CoSe–ZnSe@G) were further designed to maximize accessible active sites and improve catalytic efficiency under lean electrolyte conditions. Co/Zn‐MOFs‐nanoparticles were grown in situ on the graphene aerogel surface (Co/Zn‐MOFs@G) (**Figure** **5**a and Figure S31a, Supporting Information). A 3D CoSe–ZnSe@G derived from the Co/Zn–MOFs@G aerogel precursor was then synthesized (Figure 5b and Figure S31b, Supporting Information). The EDS analysis exhibited the uniform dispersion of C, N, Co, Zn, and Se elements (Figure 5c). XRD results showed the coexistence of graphene and CoSe–ZnSe in the as‐synthesized CoSe–ZnSe@G hybrid (Figure 5d). The highly interconnected graphene network with a macroporous structure coupled with the ultrafine nanocrystals of electrocatalytically active phase interface of CoSe–ZnSe yielded high catalytic activity and an excellent Li–S battery performance under lean electrolyte operation.

Figure 5e shows that the CoSe–ZnSe@G still delivers two typical discharge plateaus, and small voltage polarization as well as a high areal capacity of 8.0 mAh cm^−2^ at a raised sulfur loading of 7.7 mg cm^−2^ and a decreased E/S ratio of 3 µL mg^−1^. The areal capacities of 4 mAh cm^−2^ were retained at the high sulfur loading of 5.7 and 6.7 mg cm^−2^ and E/S ratio of 5 and 4 µL mg^−1^ over 50 cycles, respectively. (Figure 5f and Figure S32, Supporting Information). The decent areal capacities of 4.1 mAh cm^−2^ were retained after 40 cycles at raised sulfur loading of 7.7 mg cm^−2^ and E/S ratio of 3 µL mg^−1^ (Figure 5g). The Li–S battery with CoSe–ZnSe@G displayed a discharge capacity of 853 mAh g^−1^ at 1 C, which stabilized to around 763 mAh g^−1^ after 150 cycles under lean electrolyte condition. This corresponds to a capacity retention of 89.4% (Figure 5h), and indicates outstanding cycling stability. In terms of sulfur content, sulfur loading, areal capacity, and E/S ratio, our Li–S battery based on the CoSe–ZnSe@G design represents an important advance (Figure 5i and Table S4, Supporting Information).

## 3 Conclusion

In summary, we have shown that the enhanced bidirectional sulfur conversion by CoSe–ZnSe originated from the abundant phase boundaries with interfacial electron redistribution and lattice distortion. Both experimental and theoretical analyses demonstrated that CoSe–ZnSe heterojunction effectively immobilizes sulfur species, boosts Li ion diffusion, and decrease the energy barrier for sulfur reduction and Li_2_S decomposition in discharge and charge processes, respectively. The CoSe–ZnSe catalytic cathode gave a high areal capacity of 8.7 mAh cm^−2^, good rate capability, and excellent cyclic stability with low capacity decay rate of 0.027% per cycle. Furthermore, a 3D CoSe–ZnSe@G with an interconnected macroporous structure and fully exposed catalytic heterointerface facilitated ionic transport and bidirectional sulfur conversion reaction under high sulfur loading and lean electrolyte conditions. A high areal capacity of 8.0 mAh cm^−2^ is achieved at an E/S ratio of 3 µL mg^−1^.

## Conflict of Interest

The authors declare no conflict of interest.

## Supporting information

Supporting Information

## Data Availability

Research data are not shared.
