# Supplementary material for: Engineering Catalytic CoSe–ZnSe Heterojunctions Anchored on Graphene Aerogels for Bidirectional Sulfur Conversion Reactions
Source: Adv Sci (Weinh). 2021 Oct 27;9(1):2103456. doi: 10.1002/advs.202103456 (PMC8728854; doi:10.1002/advs.202103456)
Supplement: Supplementary file 1 — Supporting Information [file ADVS-9-2103456-s001.pdf]

WILEY-VCH

© 2021 Wiley-VCH GmbH

ADVANCED SCIENCE
Open Access

Supporting Information

for *Adv. Sci.*, DOI: 10.1002/advs.202103456

Engineering Catalytic CoSe-ZnSe Heterojunctions Anchored on Graphene Aerogels for Bidirectional Sulfur Conversion Reactions

Zhengqing Ye, Ying Jiang, Tianyu Yang, Li Li, Feng Wu, and Renjie Chen**

Supporting Information

Engineering Catalytic CoSe-ZnSe Heterojunctions Anchored on Graphene Aerogels for Bidirectional Sulfur Conversion Reactions

Zhengqing Ye, Ying Jiang, Tianyu Yang, Li Li, Feng Wu, and Renjie Chen**

Experimental Section

Preparation of bimetallic Co/Zn-MOFs. $Co(NO_3)_2{\cdot}6H_2O$ (4 mmol) and $Zn(NO_3)_2{\cdot}6H_2O$ (8 mmol) were first dissolved in methanol (160 mL). The methanol solution of 2-methylimidazole (3.94 g, 160 mL) was poured into the former mixed solution under magnetic stirring. Then, the mixture was stirred for 2 h and kept undisturbed for 4 h. The products were obtained by centrifugation, washed several times with methanol, and dried at 80 °C for 12 h. The Co-MOF and Zn-MOF were also prepared by the similar procedure, except without adding the $Zn(NO_3)_2{\cdot}6H_2O$ or $Co(NO_3)_2{\cdot}6H_2O$.

Synthesis of CoSe-ZnSe heterostructure. Typically, 100 mg Co/Zn-MOFs and 200 mg Se powder were separately put in two porcelain boats. The two porcelain boats were placed into a tube furnace with the Se powder at the upstream side. Then the furnace was heated at 800 °C under an Ar atmosphere for 3h at a ramp rate of 2 °C min^{-1}. ZnSe was also prepared according to the same process by using Zn-ZIF to replace Zn/Co-ZIFs.

Synthesis of G@CoSe-ZnSe aerogel. *Synthesis of reduced graphene oxide (rGO) aerogel.* The 3D rGO aerogel (G) was synthesized by hydrothermal reduction of

graphene oxide (GO) aqueous dispersion [1]. Briefly, 11.5 mL of 10 mg mL^{-1} GO solution and 38.5 mL water were stirred and ultrasonicated for 30 min. The obtained GO aqueous dispersion (2.3 mg mL^{-1}) was transferred into a Teflon-lined autoclave and heated at 120 °C in an oven for 24 h. The autoclave was cooled down to ambient temperature. After dialysis with deionized water/ethanol (85:15 v/v) for 2 days, and freeze-dried for 24 h to obtain G.

Synthesis of G@Co/Zn-MOFs and G@CoSe-ZnSe aerogel. 300 mg of $Co(NO_3)_2·6H_2O$ and 613 mg $Zn(NO_3)_2·6H_2O$ were dissolved in 40 mL of methanol and stirred to obtain a homogeneous solution. Then, 600 mg of polyvinylpyrrolidone (PVP MW~40,000) was added into the above solution and stirred for 10 min. The G was immersed into above solution at 30 °C, and then left standing for 12 h. Last, 40 mL of methanol with 985 mg of 2-methylimidazole quickly poured into the above solution and aged for 24 h at 30 °C. The G@Co/Zn-MOFs were obtained by washed with methanol and dried at room temperature. The preparation of the G@CoSe-ZnSe aerogel is similar to that of the CoSe-ZnSe, except that Co/Zn-MOFs are replaced by G@Co/Zn-MOFs.

Preparation of CoSe-ZnSe/S and ZnSe/S cathodes. Typically, 150 mg as-prepared CoSe-ZnSe, and 350 mg sulfur powder were uniformly mixed together then sealed in an autoclave under Ar atmosphere and heated at 155 °C for 12 h to obtain CoSe-ZnSe/S composite. The cathode was fabricated by blending the CoSe-ZnSe/S, multiwall carbon nanotube (MWCNT), and polyvinylidene fluoride (PVDF) under the mass ratio of 8:1:1 in N-methylpyrrolidone (NMP) solvent. The different areal sulfur

loading electrodes were obtained via thickness of slurry coating changed from 150 to 500 μm. The ZnSe/S cathode was prepared through the same procedure, except ZnSe/S was used as the active material instead of CoSe-ZnSe/S.

Fabrication of G@CoSe-ZnSe aerogel current collector based sulfur electrode. The G@CoSe-ZnSe current collector was prepared by mixing G@CoSe-ZnSe and the PVDF at a mass ratio of 9:1 in NMP into obtained uniform slurry, which in turn was used to coat carbon paper and dried at 60°C for 12 h. S/C slurry was prepared by infiltrating sulfur (90 wt %) into MWCNT, followed by the same slurry preparation as described above. The slurry was coated into the G@CoSe-ZnSe aerogel current collector, followed by drying at 60 ℃ for 24 h.

Material Characterization. The field-emission scanning electron microscope (FESEM) images and corresponding Energy dispersive X-ray spectroscopy (EDS) were collected by HITACHI S-4800 and EMAX350 devices. Low-resolution transmission electron microscopy (TEM), and high-resolution transmission electron microscopy (HRTEM) images were obtained with a JEOL, JEM–1200 EX microscope. High-angle annular dark field- (HAADF) images and EDS elemental mapping were carried out by using the scanning transmission electron microscopy (STEM) mode on a TECNAI G2F20. The powder X-ray diffraction (XRD) measurements were conducted on a Rigaku X-ray diffractometer (Ultima IV-185). The X-ray photoelectron spectroscopy (XPS) was carried out with a Thermo escalab 250Xi spectrometer. Nitrogen sorption measurement were performed on a Micromeritics ASAP 2460 system. Raman spectra were recorded with a Renishaw inVia

spectrometer using 532 nm wavelength for an excitation. Thermogravimetric (TGA) analysis is performed on a NETZSCH STA 449 F3. Synchrotron X-ray absorption fine structures were obtained at the beamline BL01C1 of National Synchrotron Radiation Research Center (NSRRC, Taiwan). XANES and EXAFS data were processed by standard procedures using the ATHENA program.

Adsorption and catalytic studies of CoSe-ZnSe heterointerface. Li_2S_6 solution was synthesized by mixing sulfur powder and Li_2S with a molar ratio of 5:1 in 1,2-dimethoxyethane solvent, followed by stirring for 48 h in an Ar-filled glovebox. 10 mg of CoSe-ZnSe and ZnSe powders were dispersed individually in 2 mL Li_2S_6 (5 mmol L^{-1}) solution and the hybrid were kept standing for 2 h to observe the color change. The precipitates were obtained by centrifugation and were characterized by XPS.

The catalytic property of the CoSe-ZnSe was further studied by CV and EIS tests of symmetric cells. The working electrode was prepared by mixing CoSe-ZnSe or ZnSe and PVDF at a weight ratio of 4:1 in NMP into obtained slurry, which in turn was used to coat CP and dried at 60°C. The symmetric cells were assembled by using two identical electrodes with a Celgard 2325 membrane as a separator, and 40 μL of 0.2 M Li2S8 with1 M lithium bis(trifluoromethanesulfonyl)imide (LiTFSI) electrolyte in 1:1 (v/v) DOL/DME. CV was conducted at CHI 660e electrochemical workstation at scan rate of 0.1 to 10 mV s^{-1}. EIS measurements were performed from 0.01 to 10^5 Hz.

For the Li_2S nucleation and dissolution tests, the working electrodes were

prepared by directly dropping active materials dispersions (1 mg mL^{-1}) using ethanol as solvent on the carbon paper and dried at 60°C. The Li metal as the counter electrode,20 µL of 0.2 M Li_2S_6 dissolved in 1.0 M LiTFSI with DOL/DME (v/v = 1:1) was used as catholyte, while 20 µL of identical solution as anolyte. For the Li_2S nucleation, the cells were galvanostatically discharged to 2.06 V at 0.112 mA, and then kept potentiostatically at 2.05 V until the current dropped below 10^{-5} A. For the Li_2S dissolution, the cells were galvanostatically discharged to 1.7 V at a constant current of 0.10 mA, then galvanostatically discharged to 1.7 V at 0.01 mA. Last, the cells were potentiostatically charged at 2.35 V for 20 000 s.

LSV measurements in three-electrode configuration were performed using CoSe-ZnSe or ZnSe as working electrode, Ag/AgCl electrode as the reference electrode, platinum sheet as counter electrode, and 0.1 mol L^{-1} Li_2S/methanol solution as the electrolyte. The working electrode was made by directly dropping above dispersions on the glassy carbon surface, followed by drying at 60 ℃ for 12 h. The LSV tests were conducted using electrochemical workstation from −0.8 V to −0.15 V at a scan rate of 5 mV s^{-1}.

Cell assembly and electrochemical measurements. The symmetric cell was assembled by the following process: The 90 wt% active material (CoSe-ZnSe or ZnSe) and 10 wt% PVDF binder were uniformly mixed in NMP to form a coating slurry, which was then uniformly applied to a carbon cloth. The obtained electrodes were used as the work and the counter electrode and 40 µL of Li_2S_6 (0.2 M) with 1.0 M lithium bis (trifluoromethanesulfonyl)imide (LiTFSI) in a 1:1/(v/v) 1, 3-dioxolane and

1, 2-dimethoxymethane (DOL/DME) was used as electrolyte. Standard CR2025 coin cells were fabricated in an Ar-filled glovebox, and using CoSe-ZnSe/S or ZnSe/S and Li foil as the cathode and anode, respectively. The electrolyte was composed of a mixture of 1 M LiTFSI in DOL/DME (1:1 v/v) with 0.2 M $LiNO_3$ additive. Cyclic voltammetry (CV) and electrochemical impedance spectroscopy (EIS) measurements (frequency range of 0.01 Hz to 1 MHz) were performed on CHI660E multichannel electrochemical workstation.

Theoretical Calculations.

Density functional theory (DFT) calculations were performed by the Vienna ab initio simulation package (VASP) [2], using the plane wave basis set with energy cutoff of 400 eV, the projector augmented wave (PAW) potentials, and the GGA-PBE exchange-correlation functional [3]. The surface of ZnSe (111) with four atoms layer is modeled by a supercell consisting of 3 × 3 unit cells with a vacuum region of 15 Å in the vertical, the lower half of the slabs were fixed to their bulk truncated positions allowing all other atoms to fully relax. Moreover, a heterointerface of ZnSe (111) and CoSe (110) is built. The Brillouin zone of the supercell was sampled by a 2 × 2 × 1 uniform k point mesh. The model structure with the fixed supercell was optimized using thresholds for the total energy of 10^{-5} eV and force of 0.03 eV/Å. During the structural relaxation, all of the atoms were relaxed. DFT-D3 of dispersion correction was adopted to describe the van der Waals (vdW) interactions [4]. The climbing-image nudged elastic band (CINEB) method [5] was used for the kinetic barriers of Li_2S dissociation and Li diffusion.

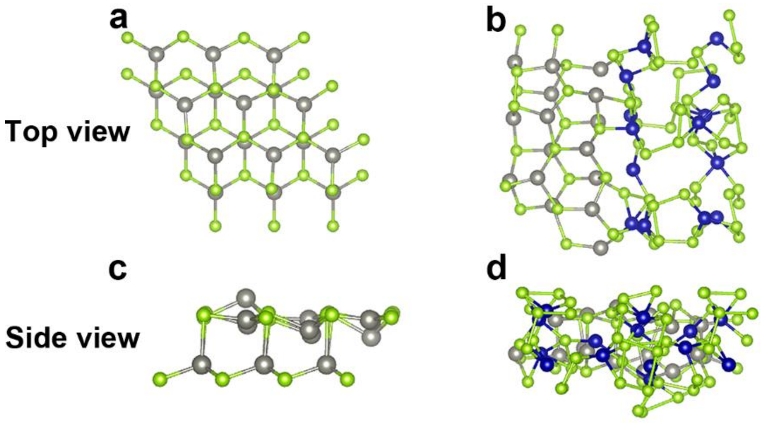

Figure S1. The structures of a,c) ZnSe and b,d) heterointerface of CoSe-ZnSe.

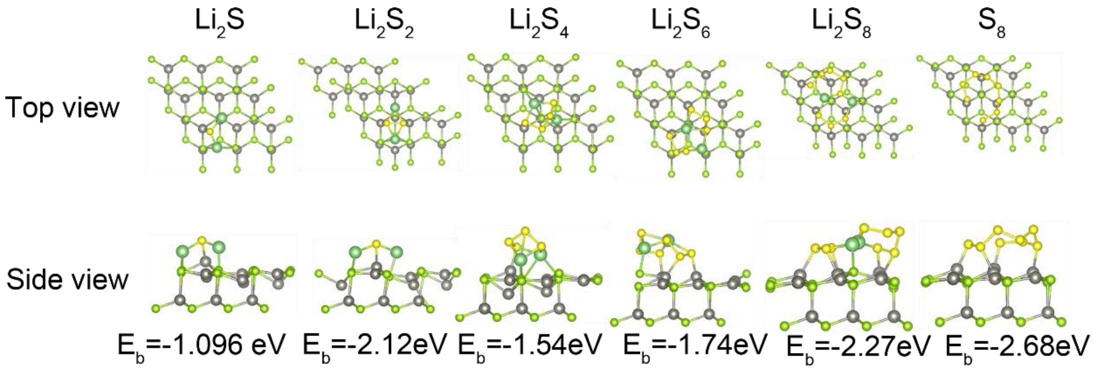

Figure S2. The optimized adsorption configurations of LiPS with ZnSe surfaces.

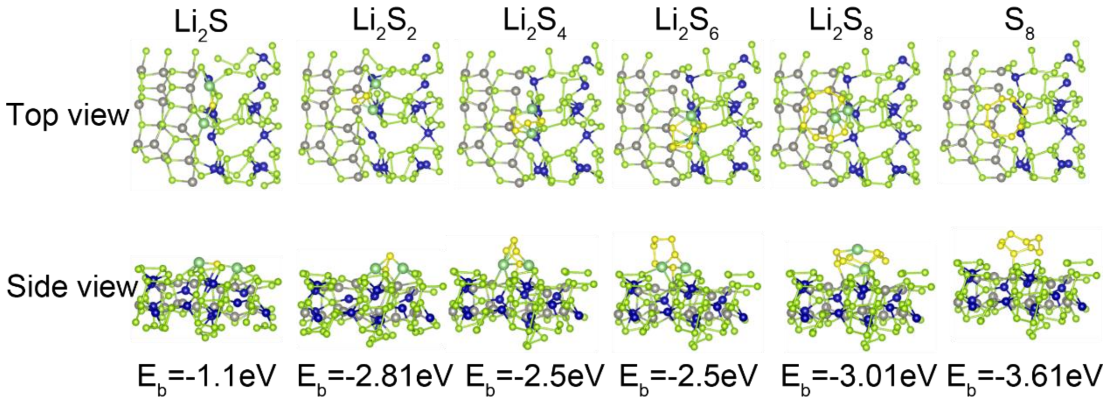

Figure S3. The optimized adsorption configurations of LiPS with CoSe-ZnSe heterointerface.

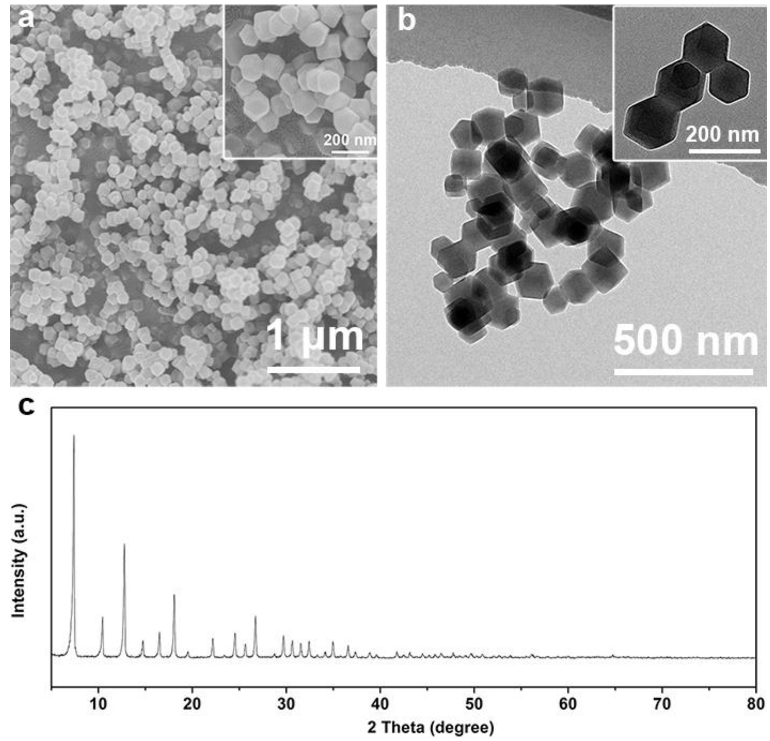

Figure S4. a) FESEM images, b) TEM images, and c) XRD pattern of bimetallic Co/Zn-MOFs.

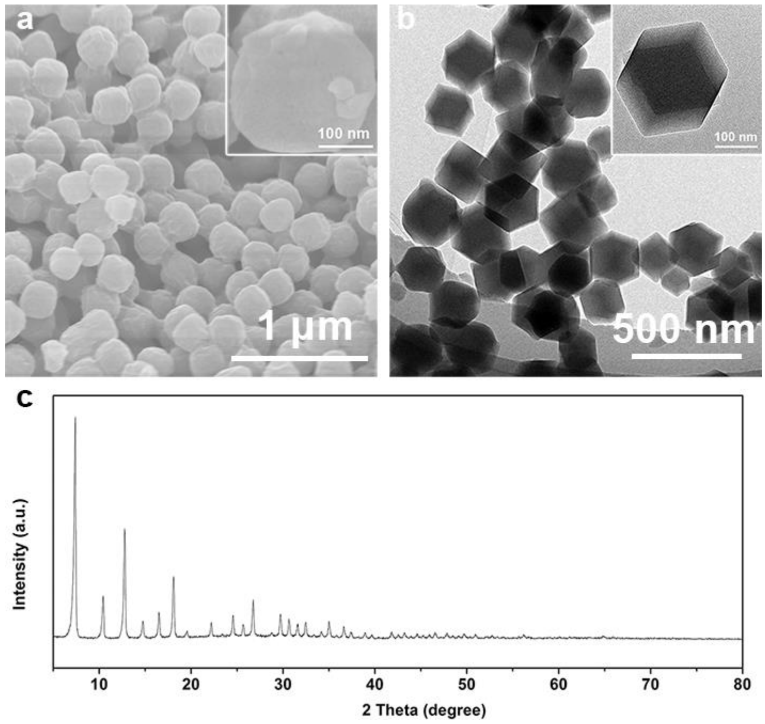

Figure S5. a) FESEM images, b) TEM images, and c) XRD pattern of Zn-MOF.

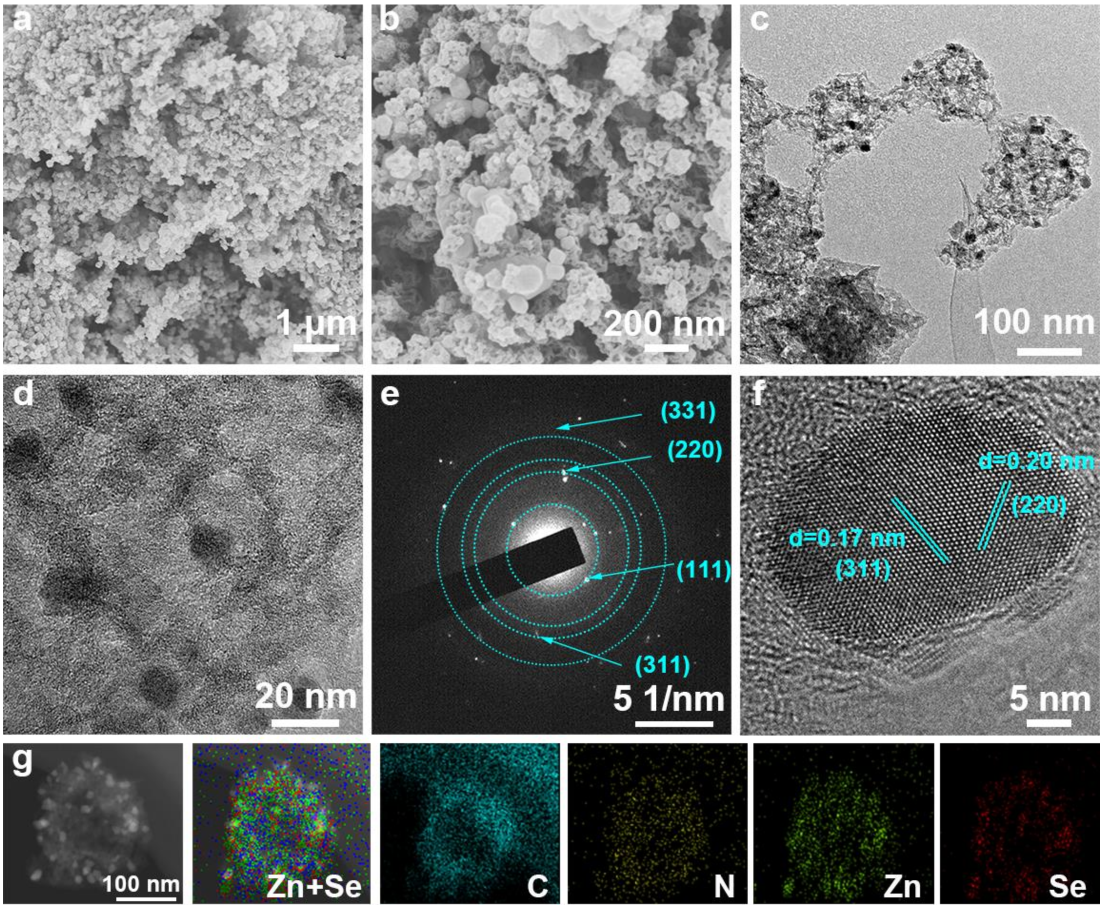

Figure S6. a,b) FESEM images, c,d) TEM image, e) SAED diffraction pattern, f) HRTEM image, and g) STEM image and corresponding EDS elemental mapping of C, N, Zn, and Se of ZnSe.

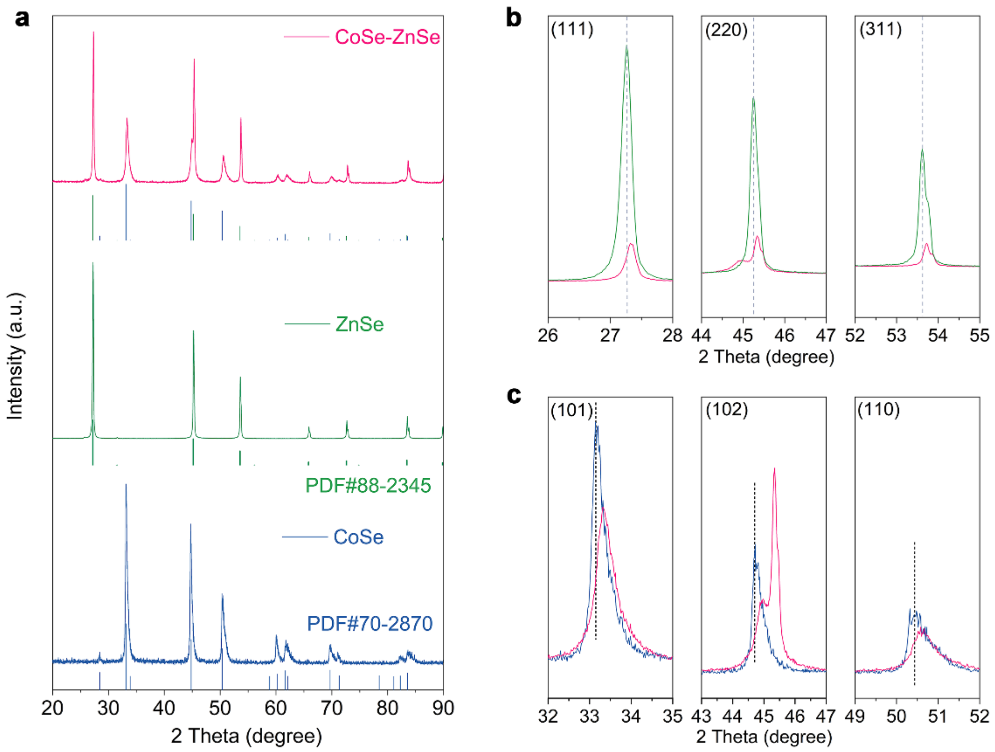

Figure S7. a-c) XRD patterns of CoSe, ZnSe, and CoSe-ZnSe.

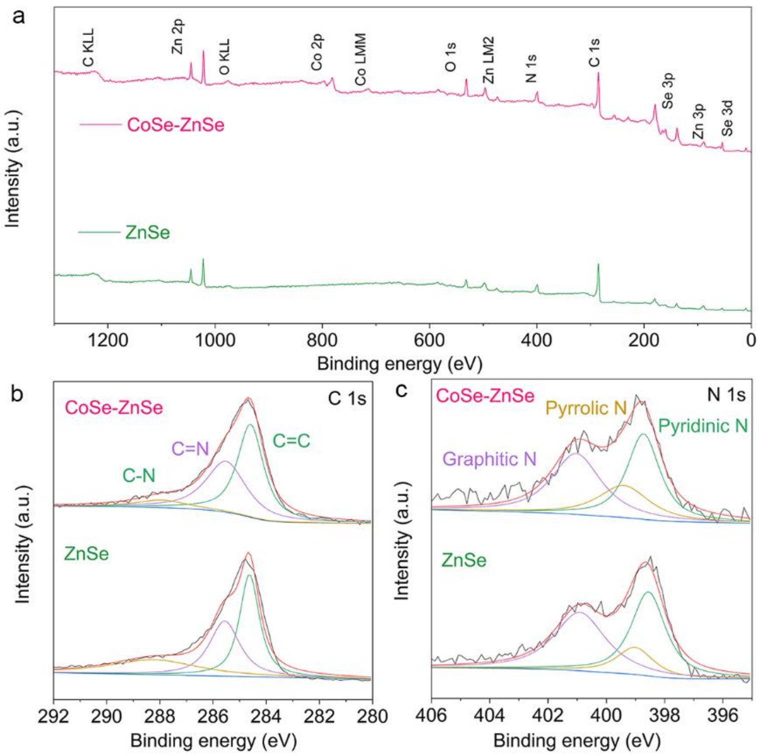

Figure S8. a) XPS survey, b) C 1s, and c) N 1s spectra of CoZn-ZnSe and ZnSe respectively. The C 2p spectrum in Figure S8b can be fitted into three peaks corresponding to C=C (284.59+0.04 eV), C=N (285.52+0.04 eV), and C−N (287.91+0.3 eV). As shown in the Figure S8c. N 1s spectra can be deconvoluted into three characteristic peaks: pyridinic N (398.58-398.72 eV), pyrrolic N (399.01-399.4 eV), and graphitic N (400.91-401.03 eV).

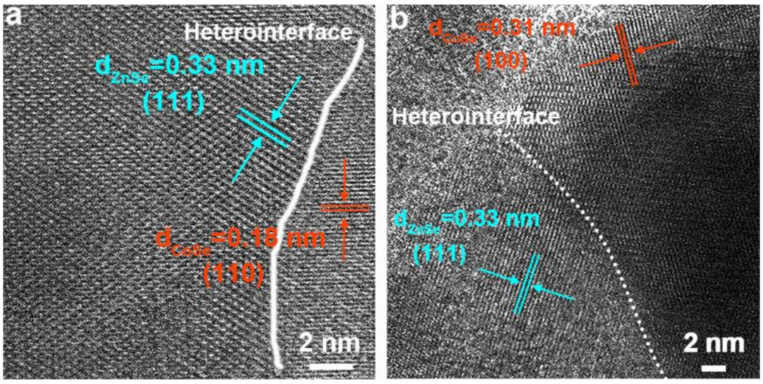

Figure S9. HRTEM images of CoSe-ZnSe.

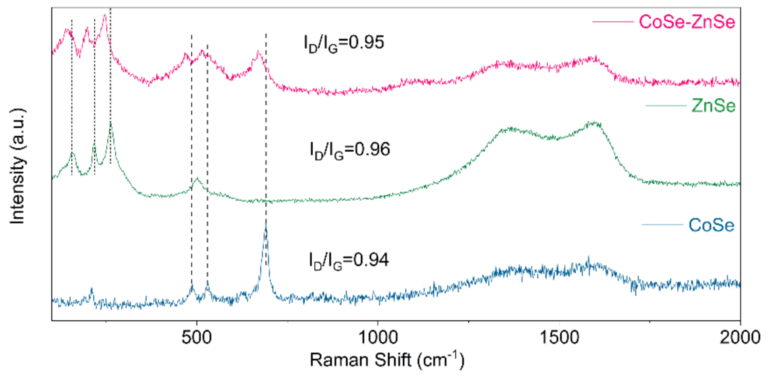

Figure S10. Raman spectra of CoSe, ZnSe, and CoSe-ZnSe, respectively.

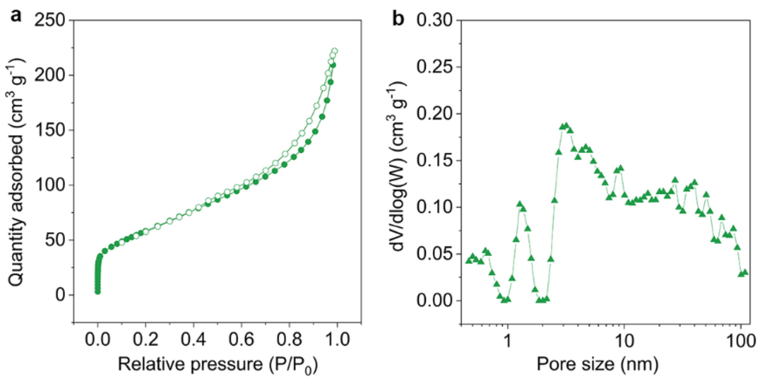

Figure S11. a) N_2-asorption isotherms and b) pore size distribution of ZnSe.

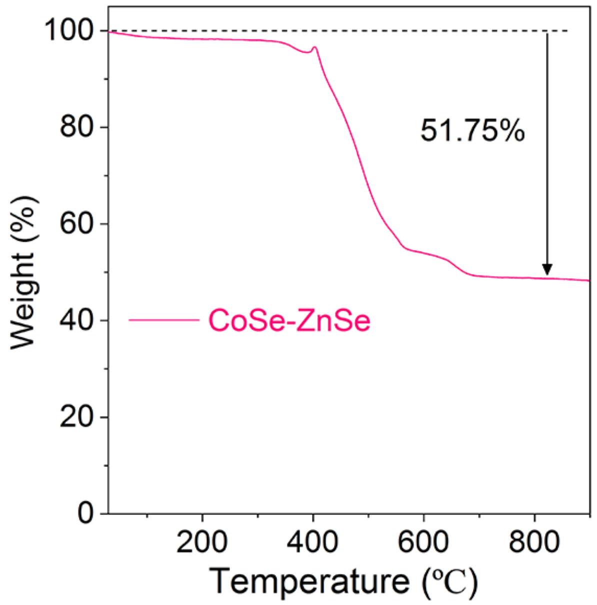

Figure S12. TGA curves of the as-prepared CoSe-ZnSe sample at a ramp rate of 10 °C min^{-1} under air atmosphere.

When the CoSe-ZnSe sample was heated at the temperature range of 27 to 900 °C, the phases of the components in the heterostructure were converted according to the following three chemical reactions:

$C (s) + O_2 (g) = CO_2 (g)$ (S1)

$2ZnSe (s) + 3O_2 (g) = 2ZnO (s) + 2SeO_2 (g)$ (S2)

$3CoSe (s) + 5O_2 (g) = Co_3O_4 (s) + 3SeO_2 (g)$ (S3)

The ratio of Zn and Co in the CoSe-ZnSe is 1.18. Based on the above chemical reaction (S1-S3), the contents of the CoSe, ZnSe, and carbon are calculated to be 38.02 wt.%, 46.32 wt.%, 15.66 wt.%, respectively.

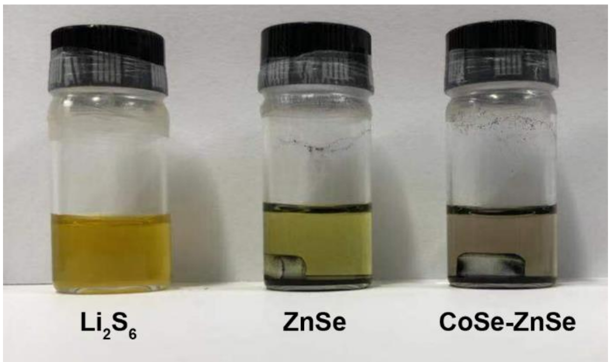

Figure S13. Adsorption ability tests of ZnSe and CoSe-ZnSe with 5 mM Li_2S_6 solution into 2 mL of DME containing ZnSe and CoSe-ZnSe powder with the same weight (10 mg).

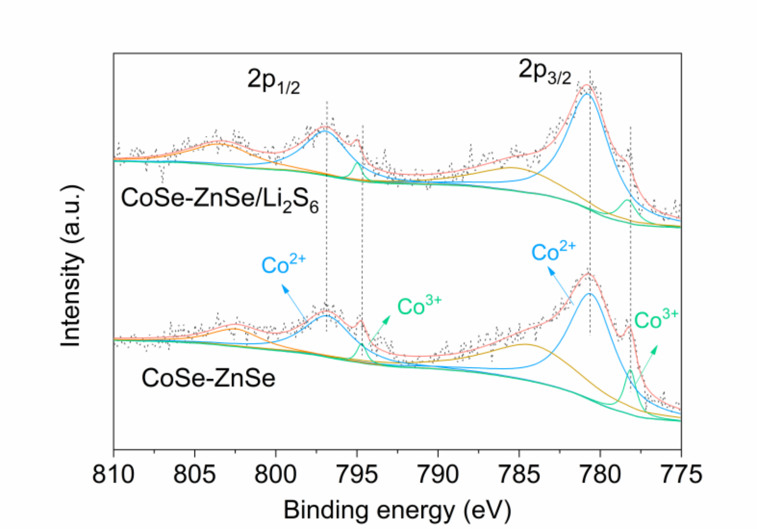

Figure S14. High-resolution XPS spectra of Co 2p of CoSe-ZnSe heterostructure before and after adsorption of Li_2S_6.

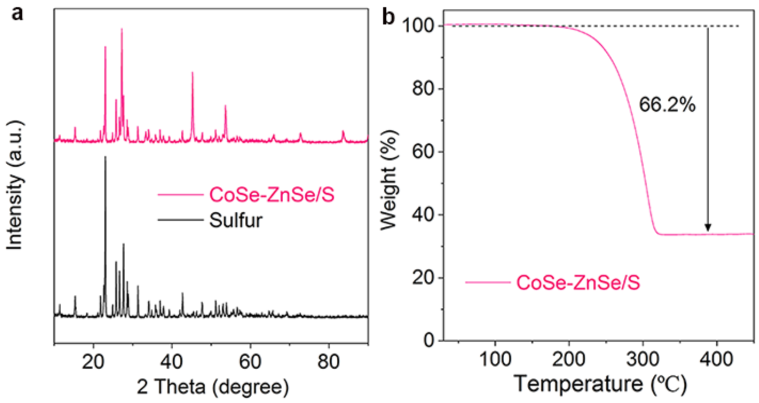

Figure S15. a) XRD patterns and b) TGA curve of CoSe-ZnSe/S.

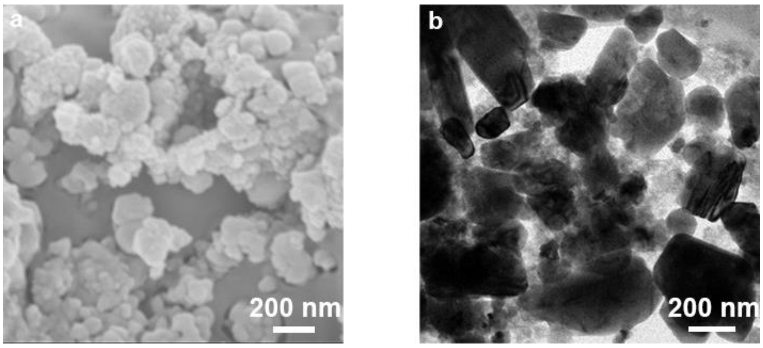

Figure S16. a) FESEM images and b) TEM image of CoSe-ZnSe/S.

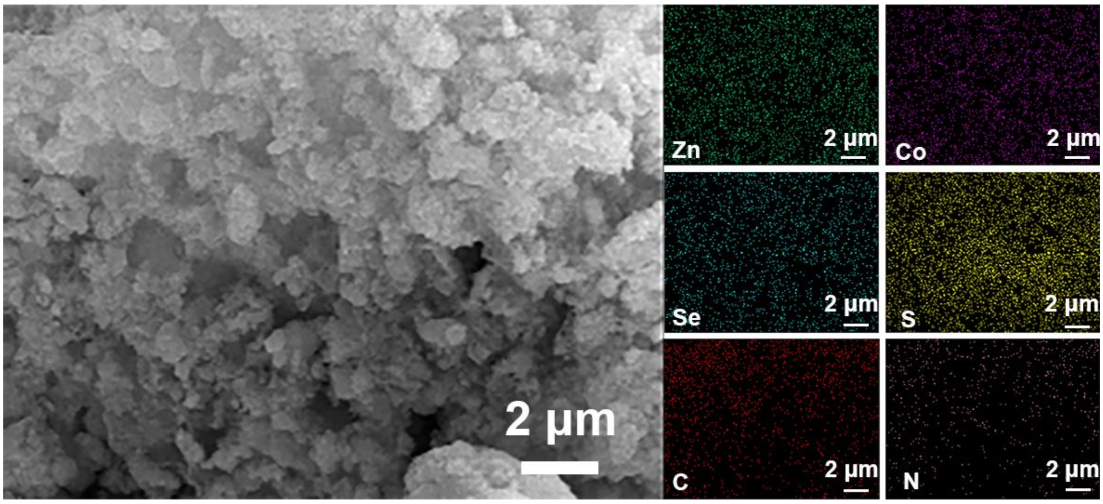

Figure S17. FESEM image of CoSe-ZnSe/S and corresponding EDS elemental mapping of Co, Zn, Se, S, C, and N

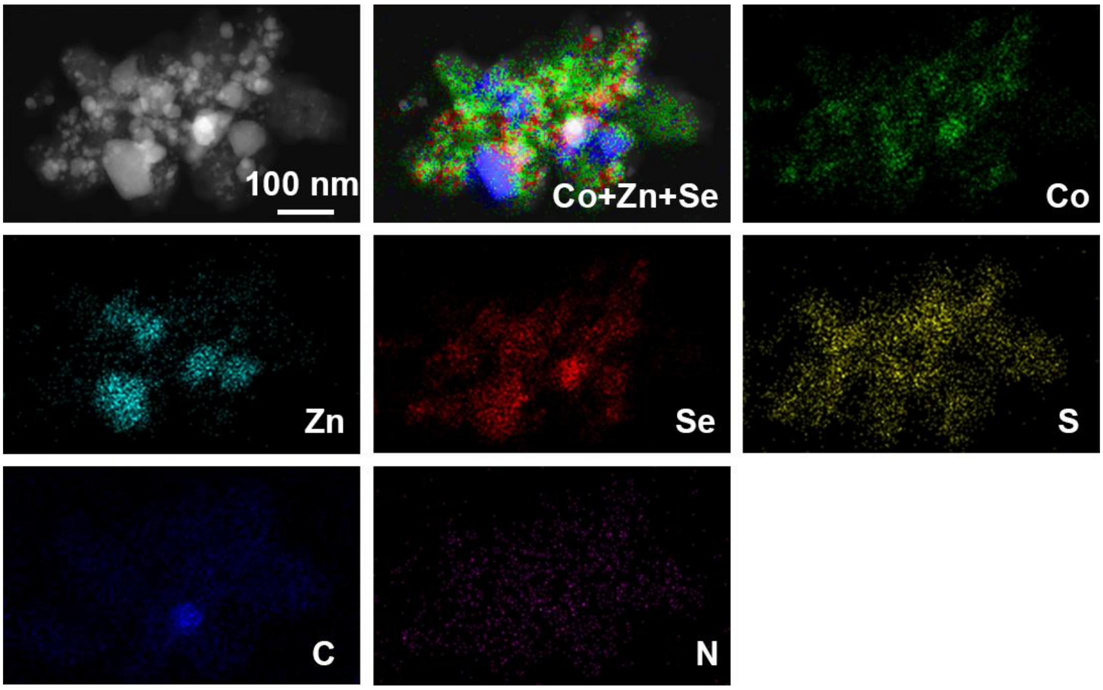

Figure S18. HAADF-STEM image of CoSe-ZnSe/S and corresponding EDS elemental mapping of Co, Zn, Se, S, C, and N.

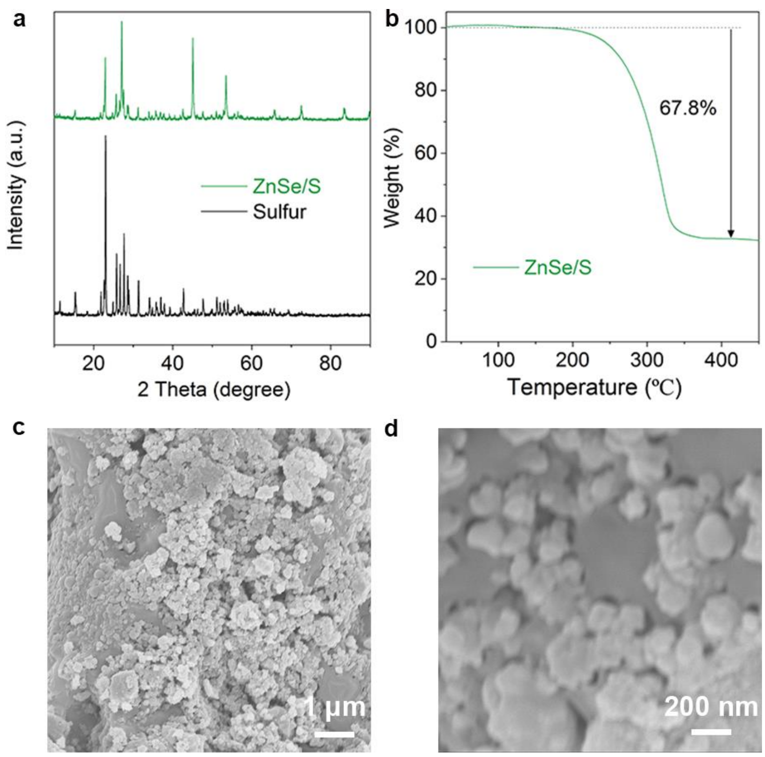

Figure S19. a) XRD patterns, b) TGA curve, and c,d) FESEM images of ZnSe/S.

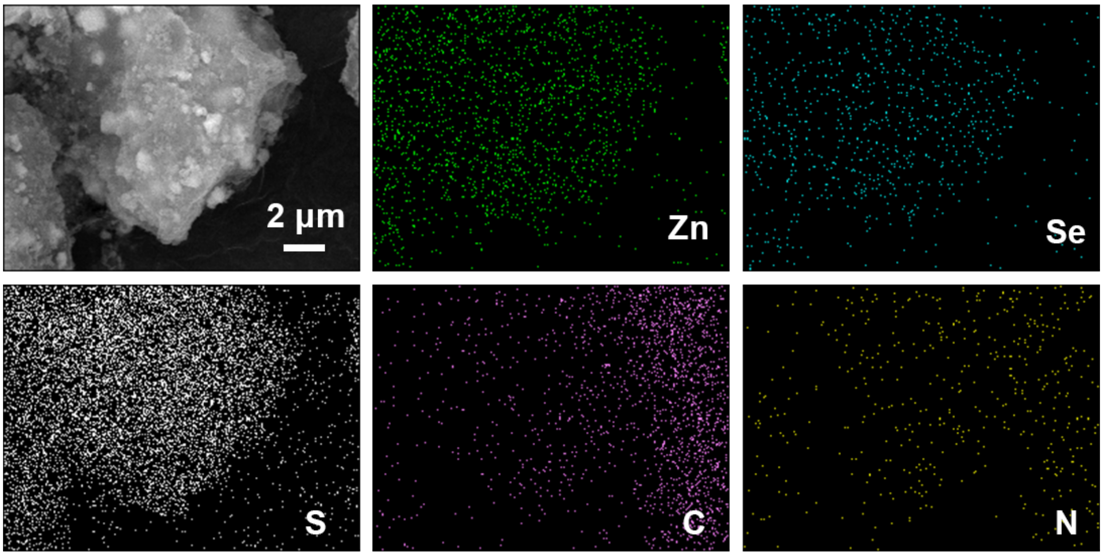

Figure S20. FESEM image of ZnSe/S and corresponding EDS elemental mapping of Zn, Se, S, C, and N.

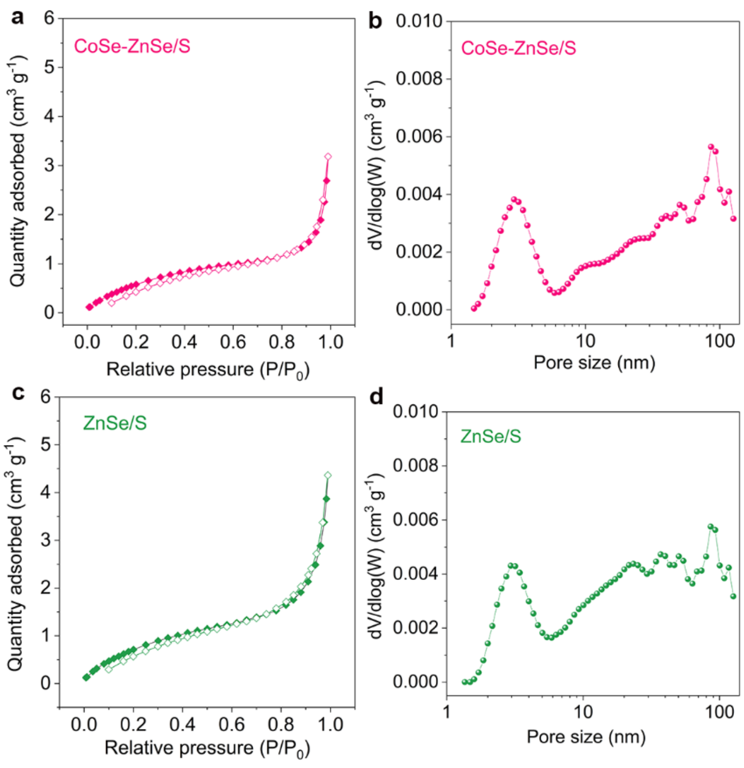

Figure S21. a,c) N_2 physisorption isotherms and b,d) pore size distribution curves of CoSe-ZnSe/S and ZnSe/S, respectively.

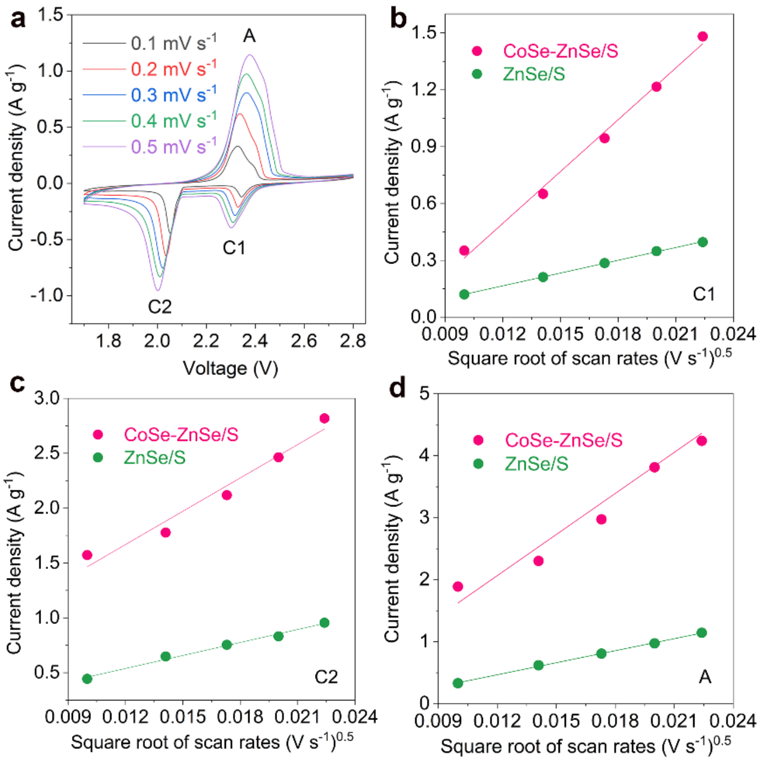

Figure S22. a) CV curves of the ZnSe/S electrode at various voltage scan rates. Plot of CV peak current versus square root scan rates for b,c) the cathodic reactions and d) the anodic reaction.

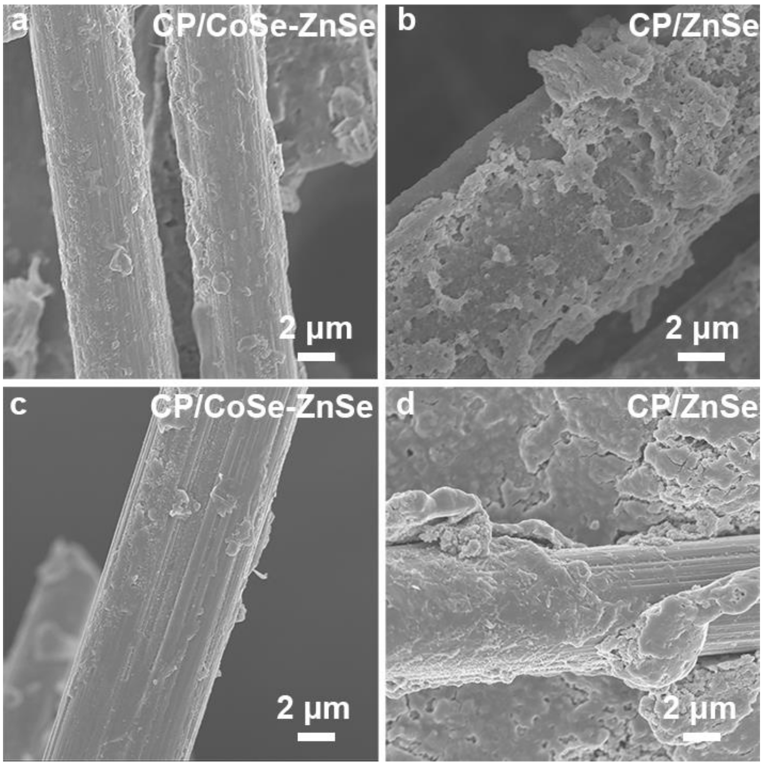

Figure S23. SEM images showing the morphology of a,b) Li_2S nucleation and c,d) dissolution on different surfaces.

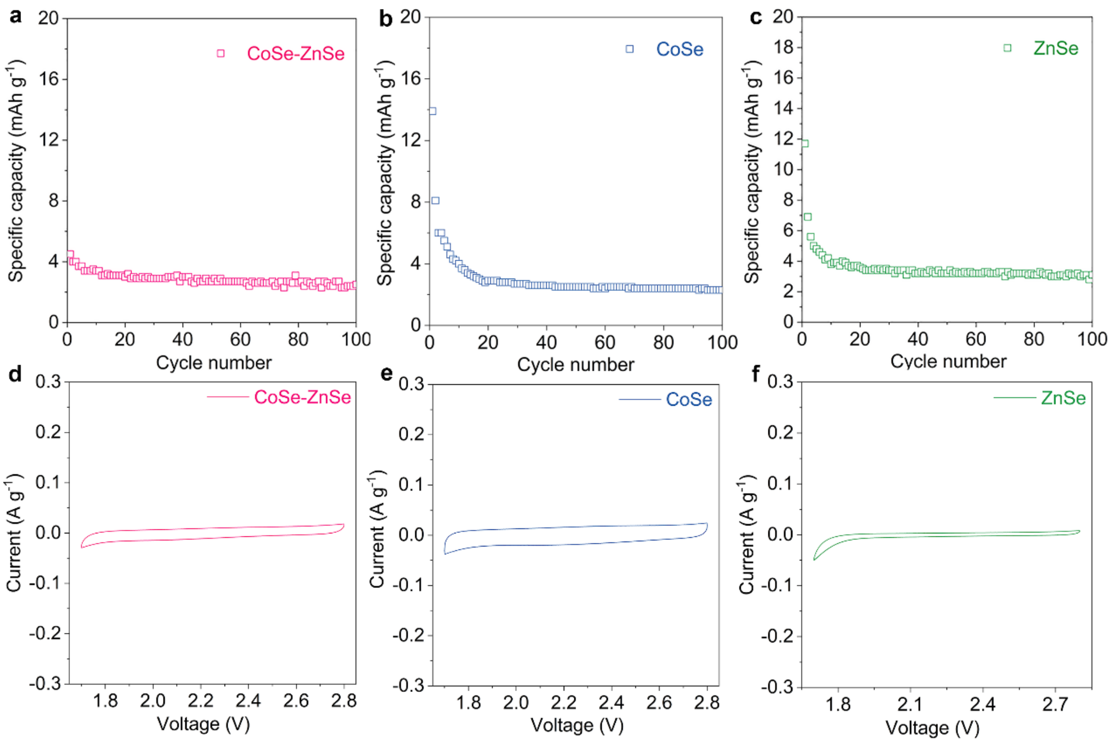

Figure S24. Cycling performance of a) CoSe-ZnSe, b) CoSe, and c) ZnSe electrodes at 0.2 C within a potential window of 1.7–2.8 V vs. Li/Li^+. CV curves of d) CoSe-ZnSe, e) CoSe, and f) ZnSe electrodes at a scan rate of 0.2 mV s^{-1} within a potential window of 1.7–2.8 V vs. Li/Li^+.

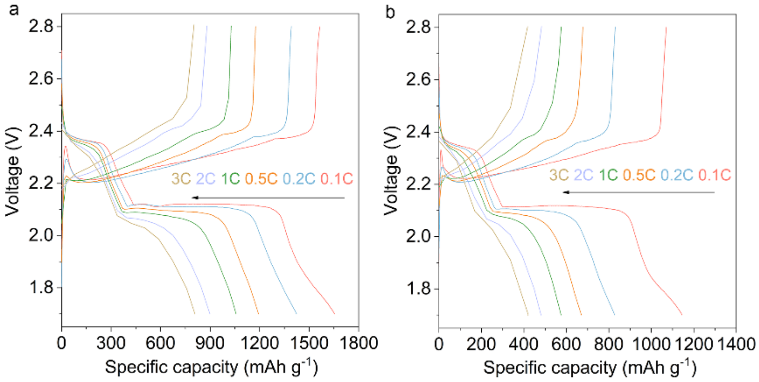

Figure S25. Multi-rate discharge-charge profiles of a) CoSe-ZnSe/S and b) ZnSe/S.

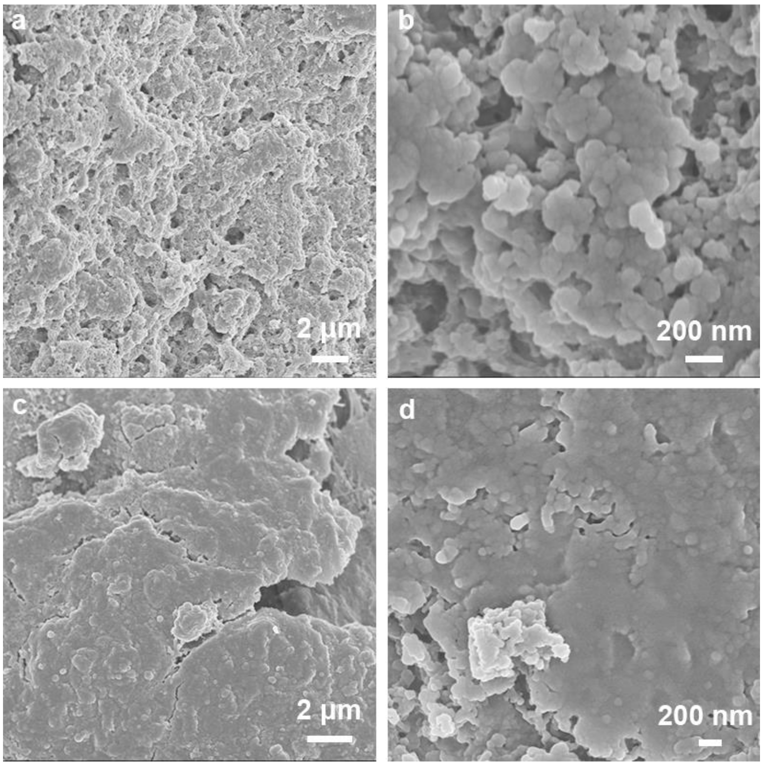

Figure S26. FESEM images of a,b) CoSe-ZnSe/S and c,d) ZnSe/S electrodes after 100 cycles at 0.2 C.

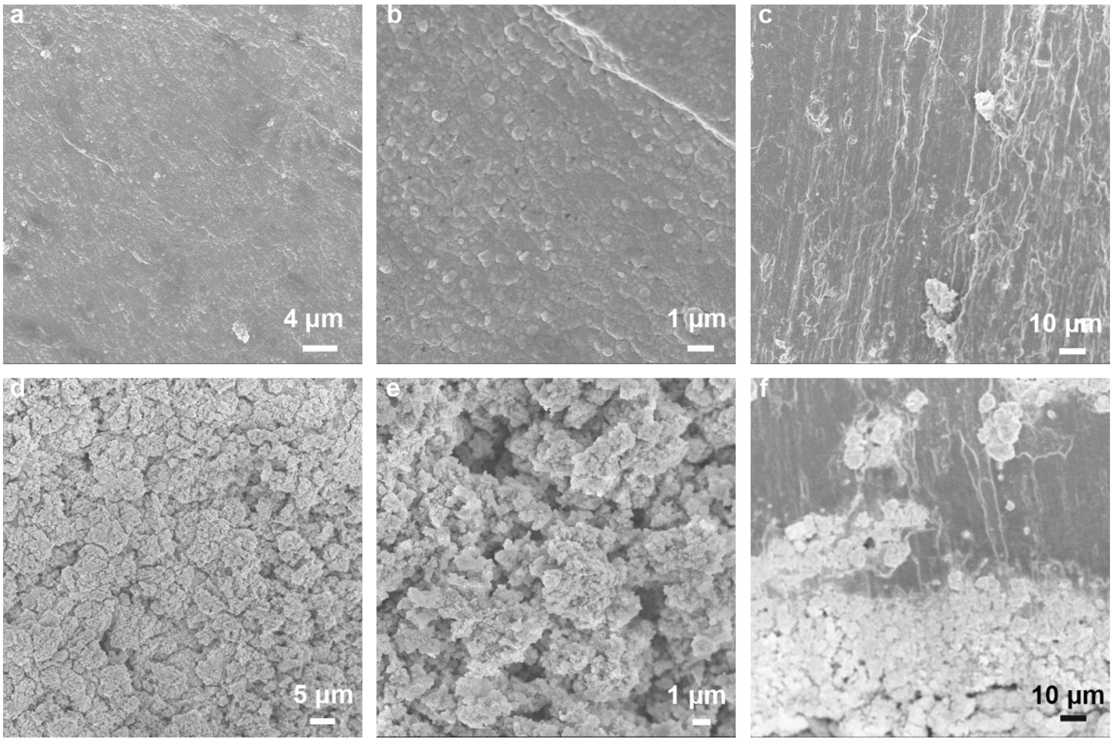

Figure S27. FESEM images of Li metal surface form the a,b) CoSe-ZnSe/S and d,e) ZnSe/S electrodes after 100 cycles 0.2 C. Cross-sectional view FESEM images of Li metal form the c) CoSe-ZnSe/S and f) ZnSe/S electrodes after 100 cycles 0.2 C.

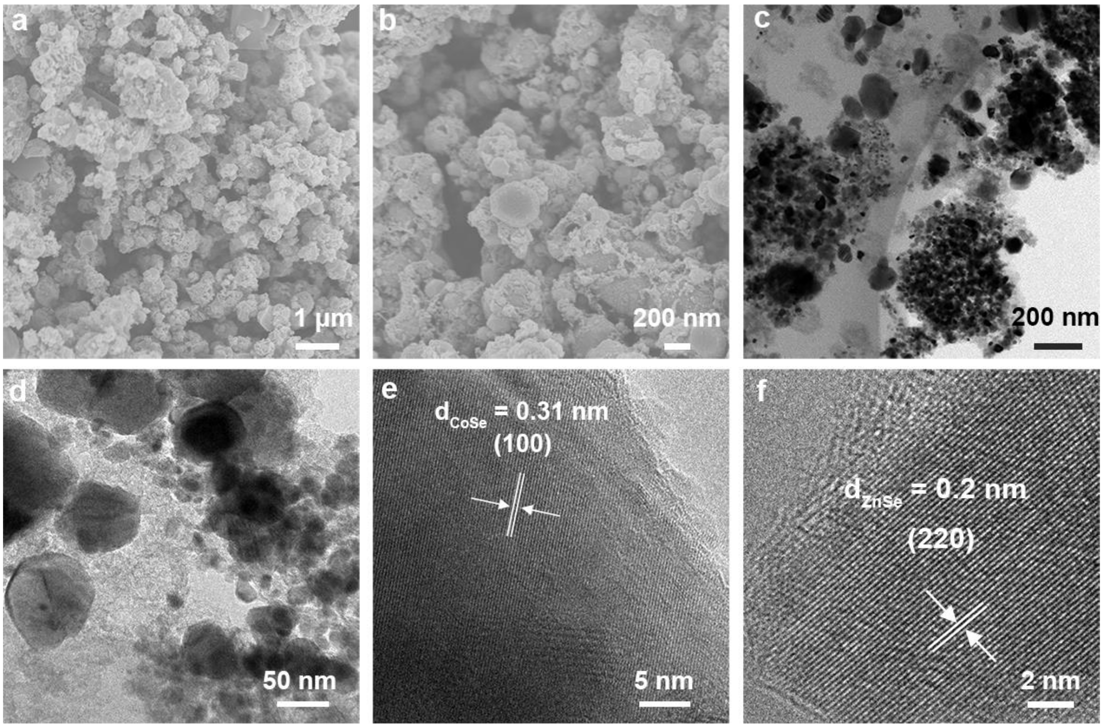

Figure S28. a,b) FESEM images, c,d) TEM images, and e,f) HRTEM image of CoSe/ZnSe hybrid.

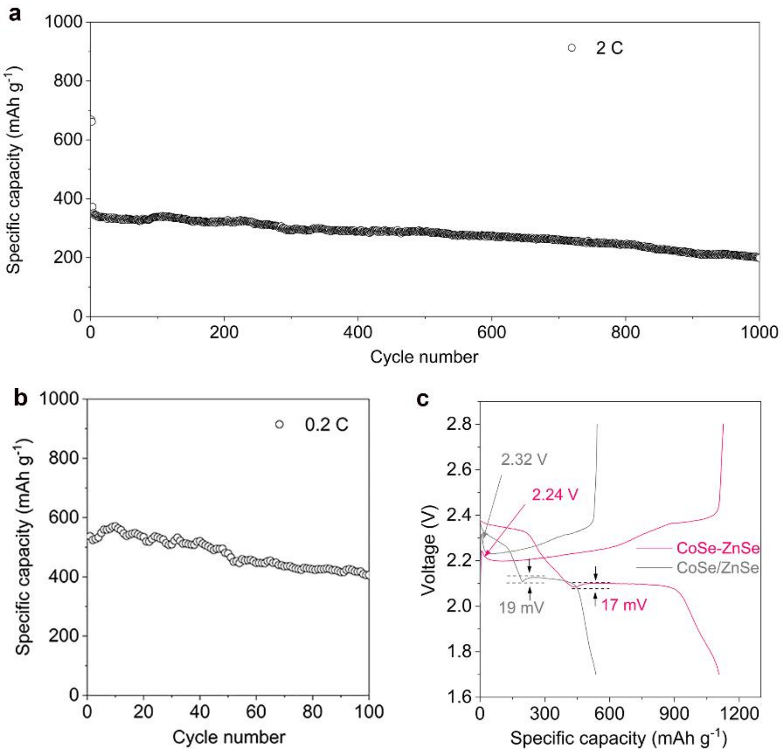

Figure S29. a,b) Cycle performance of CoSe/ZnSe sulfur cathode. c) discharge/charge curves at 0.2 C.

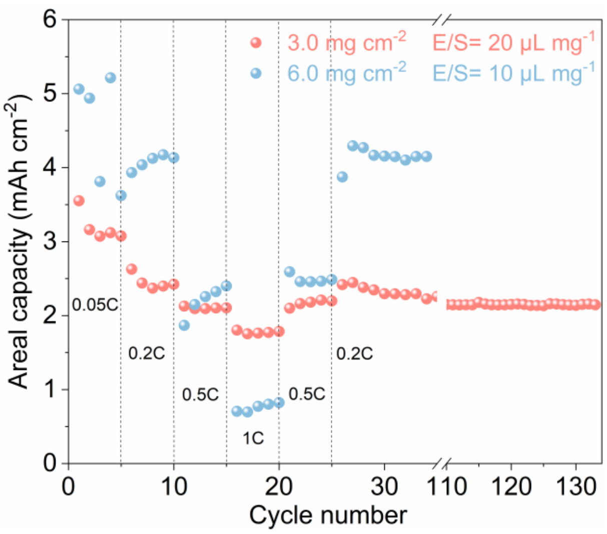

Figure S30. Cycle and rate capability of CoSe-ZnSe/S electrodes with different sulfur loading and E/S ratio.

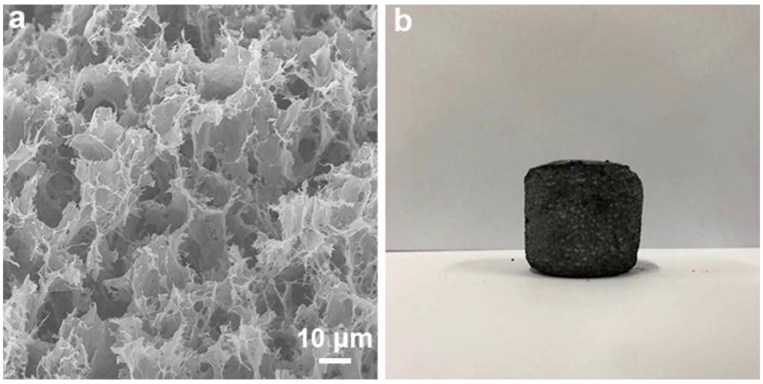

Figure S31. a) SEM image of G. b) photograph of a CoSe-ZnSe@G aerogel.

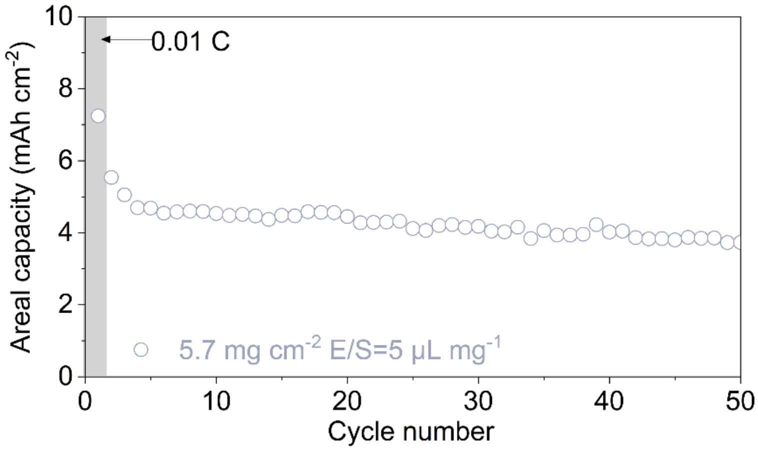

Figure S32. The cycling performance of Li-S batteries with G@CoSe-ZnSe at 0.05 C.

Table S1. The specific surface area and pore structure of obtained samples.

Sample	S_{BET} a) (m^2 g^{-1})	$S_{micropore}$ b) (m^2 g^{-1})	V_t c) (cm^3 g^{-1})	$V_{micropore}$ d) (cm^3 g^{-1})
CoSe-ZnSe	223	36	0.37	0.02
ZnSe	213	46	0.34	0.03

a)Total specific surface area, determined by the BET method; b)The specific surface area of the micropore, calculated by the t-Plot method; c)Total pore volume, examined at P/P_0 of 0.98; d)The pore volume of the micropore, determined by the t-Plot method.

Table S2.EXAFS fitting parameters at the Zn K-edge for various samples（S_0^2=0.89）

sample	shell	CN	R(Å)	σ^2	ΔE_0	R factor
ZnO	Zn-O	4	1.96±0.01	0.0046	0.9±0.1	0.0022
ZnSe	Zn-Se	4.7±0.3	2.45±0.01	0.0061	3.6±0.8	0.0031
CoSe-ZnSe	Zn-Se	3.4±0.7	2.45±0.01	0.0053	3.4±0.7	0.0121

^aN: coordination numbers; ^bR: bond distance; $^c\sigma^2$: Debye-Waller factors; ^{d} ΔE_0: the inner potential correction. *R* factor: goodness of fit. S_0^2 was set to 0.89, according to the experimental EXAFS fit of ZnO reference by fixing CN as the known crystallographic value; δ: percentage.

Table S3. Comparison of S_{BET} and electrochemical performance of state-of-the-art transition metal based heterostructure sulfur host.

Host material	S_{BET} (m^2 g^{-1})	Capacity (mAh g^{-1}) (Low rate)	Capacity (mAh g^{-1}) (High rate)	Decay rate (per cycle, %) (cycles, rate)	Areal Capacity (mAh cm^{-2}) (rate)	S content (wt%)	Ref
**CoSe-ZnSe**	**222.56**	**1654 (0.1 C)**	**808 (3C)**	**0.029% (1700, 2 C)**	**8.7 (0.01 C) 5.6 (0.1 C)**	**66.2**	**This work**
MoN-VN	-	1436 (0.1 C)	708 (2 C)	0.068% (500, 2 C)	3.8 (0.1 C)	58.5	[6]
VO_2-VN	46	1425 (0.2 C)	935 (2 C)	0.06% (800, 2 C)	4.7 (0.3 C)	61.8	[7]
NiO-$NiCo_2O_4$	-	1063.2 (0.2 C)	697.9 (2 C)	0.059% (500, 0.5 C)	-	73	[8]
MoO_2-Mo_3N_2	94.87	900 (0.5 C)	448 (3 C)	0.032% (1000, 1 C)	-	75	[9]
Fe_2O_3@Mn_3O_4	21.22	1321 (0.2)	971 (2 C)	0.076% (100, 0.1 C)	5.08 (0.1 C)	70.1	[10]
VTe_2@MgO	17.8	1160 (0.2 C)	705 (3 C)	0.055% (1000, 1 C)	5.3 (0.2 C)	60	[11]
ZnS-FeS	186.78	1278 (0.2 C)	718 (4 C)	0.17% (200, 0.2 C)	-	69.93	[12]
$MoSe_2$/MoO_2	104	1205 (0.1 C)	874 (2 C)	0.046% (500, 0.5 C)	6.79 (0.1 C)	66	[13]
TiO_2-Ni_3S_2/rGO	-	1270 (0.1 C)	534 (5 C)	0.038% (900, 0.5 C)	-	64	[14]
V_8C_7-VO_2	141.1	1294 (0.2 C)	643.5 (6 C)	0.061% (900, 4 C)	-	62.8	[15]

Table S4. Comparison of electrochemical performance of TMSe-based hybrid for Li-S batteries.

TMSe-based hybrid	Capacity (mAh g^{-1}) (Low rate)	Capacity (mAh g^{-1}) (High rate)	Decay rate (per cycle, %) (cycles, rate)	Areal Capacity (mAh cm^{-2}) (E/S ratio)	Ref
**CoSe-ZnSe@G**	**1654 (0.1 C)**	**808 (3C)**	**0.029% (1700, 2 C)**	**8.0 (3 μL mg^{-1})**	**This work**
CC@CS @HPP	1634.9 (0.1 C)	754.3 (3 C)	0.04% (1200, 2 C)	8.7 (7 μL mg^{-1})	[16]
CoSe/C HSs	1405 (0.1 C)	913.7 (5 C)	0.034% (500, 1 C)	8.8 (3 μL mg^{-1})	[17]
CC/ $MoSe_2$	1411 (0.1 C)	872 (2 C)	0.038% (500, 1 C)	5.5 (6.3 μL mg^{-1})	[18]
ZnSe/N-C	1475 (0.1 C)	542 (3 C)	0.022% (800, 1 C)	3.2 (15.6 μL mg^{-1})	[19]
Co-$NiSe_2$ @NC	903.3 (0.5 C)	598.9 (2 C)	-	-	[20]
G-Sb_2Se_{3-x}	1387 (0.1 C)	787 (8 C)	0.027% (500, 1 C)	7.5 (10 μL mg^{-1})	[21]
$MoSe_2$/ $Ti_3C_2T_x$	1454 (0.1 C)	759 (5 C)	0.095% (500, 0.5 C)	8.0 (3.5 μL mg−1)	[22]
$MoSe_2$@ rGO	1608 (0.1 C)	863 (2 C)	0.042% (250, 0.25 C)	-	[23]
RGO-$CoSe_2$	1044.7 (0.2 C)	695.7 (2 C)	0.071% (400, 1 C)	-	[24]
$FeSe_2$@C	1341 (0.1 C)	767 (3 C)	0.04% (700, 1 C)	-	[25]
FeSe@ NC	1371 (0.1 C)	447 (5 C)	0.1% (400, 1 C)	-	[26]

Reference:

[1] C. Li, J. Yang, P. Pachfule, S. Li, M. Y. Ye, J. Schmidt, A. Thomas, *Nat. Commun.* **2020**, 11, 4712.

[2] P. Hohenberg, W. Kohn, *Phys. Rev. B* **1964**, 136, B864.

[3] J. P. Perdew, J. A. Chevary, S. H. Vosko, K. A. Jackson, M. R. Pederson, D. J. Singh, C. Fiolhais, *Phys. Rev. B* **1992**, 46, 6671.

[4] J. Klimes, D. R. Bowler, A. Michaelides, *Phys. Rev. B* **2011**, 83, 195131.

[5] G. Henkelman, B. P. Uberuaga, H. Jonsson, *J. Chem. Phys.* **2000**, 113, 9901.

[6] c. ye, y. jiao, h. jin, A. Slattery, K. Davey, h. wang, S. Qiao, *Angew. Chem. Int. Ed.* **2018**, 57, 16703.

[7] Y. Z. Song, W. Zhao, L. Kong, L. Zhang, X. Y. Zhu, Y. L. Shao, F. Ding, Q. Zhang, J. Y. Sun, Z. F. Liu, *Energy Environ. Sci.* **2018**, 11, 2620.

[8] H. Linyu, D. Chunlong, L. Heng, L. Yi, S. Bolei, C. Yuming, B. Shu- Juan, X. Maowen, *Adv. Energy Mater.* **2018**, 8, 1800709.

[9] R. Li, X. Zhou, H. Shen, M. Yang, C. Li, *ACS Nano* **2019**, 13, 10049.

[10] H. Liu, Z. Chen, L. Zhou, K. Pei, P. Xu, L. Xin, Q. Zeng, J. Zhang, R. Wu, F. Fang, R. Che, D. Sun, *Adv. Energy Mater.* **2019**, 9, 1901667.

[11] M. Wang, Y. Song, Z. Sun, Y. Shao, C. Wei, Z. Xia, Z. Tian, Z. Liu, J. Sun, *ACS Nano* **2019**, 13, 13235.

[12] W. Li, Z. Gong, X. Yan, D. Wang, J. Liu, X. Guo, Z. Zhang, G. Li, *J. Mater. Chem. A* **2020**, 8, 433.

[13] Q. Hao, G. Cui, Y. Zhang, J. Li, Z. Zhang, *Chem. Eng. J.* **2020**, 381, 122672.

[14]R. Wang, C. Luo, T. Wang, G. Zhou, Y. Deng, Y. He, Q. Zhang, F. Kang, W. Lv, Q.-H. Yang, *Adv. Mater.* **2020**, 32, 2000315.

[15]J. Cai, J. Jin, Z. Fan, C. Li, Z. Shi, J. Sun, Z. Liu, *Adv. Mater.* **2020**, 32, 2005967.

[16]Z. Ye, Y. Jiang, L. Li, F. Wu, R. Chen, *Adv. Mater.* **2020**, 32, 2002168.

[17]S. Hu, Y. Hu, X. Liu, J. Zhang, *Nanoscale* **2021**, 13, 10849.

[18]H. Yang, M. S. Wang, T. Wang, H. Xu, Z. L. Yang, L. Zhang, J. C. Chen, Y. Huang, X. Li, *Int. J. Electrochem. Sci.* **2020**, 15, 7585.

[19]D. Yang, C. Zhang, J. J. Biendicho, X. Han, Z. Liang, R. Du, M. Li, J. Li, J. Arbiol, J. Llorca, Y. Zhou, J. R. Morante, A. Cabot, *ACS Nano* **2020**, 14, 15492.

[20]T. M. Chen, Z. C. Shang, B. Yuan, N. X. Wu, M. Abuzar, J. Y. Yang, X. X. Gu, C. Y. Miao, M. Ling, S. Li, *Energy Technol.* **2020**, 8, 7.

[21]Y. Tian, G. Li, Y. Zhang, D. Luo, X. Wang, Y. Zhao, H. Liu, P. Ji, X. Du, J. Li, Z. Chen, *Adv. Mater.* **2019**, 1904876.

[22]W. Wang, L. Huai, S. Wu, J. Shan, J. Zhu, Z. Liu, L. Yue, Y. Li, *ACS Nano* **2021**, 15, 11619.

[23]W. Tian, B. Xi, Z. Feng, H. Li, J. Feng, S. Xiong, *Adv. Energy Mater.* **2019**, 9, 1901896.

[24]L. Chen, W. Yang, J. Liu, Y. Zhou, *Nano Res.* **2019**, 12, 2743.

[25]W. Sun, Y. Li, S. Liu, C. Liu, X. Tan, K. Xie, *Chem. Eng. J.* **2021**, 416, 129166.

[26]J. Li, X. Niu, P. Zeng, M. Chen, Y. Pei, L. Li, Z. Luo, X. Wang, *Chem. Eng. J. Chem. Eng. J.* ***2021***, 421, 129770.
